# Inverse scattering of absorptive samples via beam propagation

**DOI:** 10.1364/optica.599869

**Published:** 2026-09-11

**Authors:** Peter Wagenaar, Jeongsoo Kim, Mary E. Swartz, Johann K. Eberhart, Shwetadwip Chowdhury

**Affiliations:** 1 Department of Electrical and Computer Engineering, University of Texas at Austin, 2501 Speedway, Austin, Texas 78712, USA; 2 Department of Molecular Biosciences, University of Texas at Austin, 2501 Speedway, Austin, Texas 78712, USA

## Abstract

Inverse-scattering methods enable label-free, quantitative visualization of a sample’s three-dimensional (3D) refractive index (RI), providing intrinsic volumetric contrast. Recent advances have demonstrated successful 3D RI reconstructions in multiple-scattering samples using angle-varying illumination; however, these studies have primarily focused on *non-absorptive* samples. Here, we extend the multi-slice beam propagation (MSBP) inverse-scattering framework to reconstruct *complex-valued* RI, encompassing both the sample’s conventional RI (real part) and absorptivity (imaginary part). We show that reconstructing complex-valued RI makes the inverse problem ill-posed under angle-varying illumination alone, and that incorporating measurement diversity from both angle-varying illumination and sample defocus enables stable and accurate convergence. Experimental demonstrations were conducted on (1) dyed microsphere samples to characterize the accuracy of the reconstructed RI and absorptivity and (2) diverse scattering and absorptive bio-samples to demonstrate biological utility. These results represent an important step for label-free volumetric imaging in biological tissue, which typically exhibits *both* scattering *and* absorption.

## 1. INTRODUCTION

Optical imaging is fundamentally constrained by scattering, which can quickly degrade imaging quality in biological tissue starting at depths of just a few tens of microns. Inverse-scattering methods seek to address this challenge by computationally undoing scattering effects to reconstruct a sample’s volumetric refractive index (RI), which provides label-free quantitative morphological contrast. Traditional methods for 3D RI reconstruction have relied on *weak-scattering* approximations, such as the first Born [1] or Rytov [2], to linearize the relationship between the sample’s 3D RI and its scattering measurements [3–5]. This approach allows efficient and analytical reconstruction of the sample’s RI and has enabled a wide range of applications, including studies of cell morphology [6–8], developmental processes [9], pathology [10–13], etc.

However, because such approaches rely on the weak-scattering assumption, they are generally limited to imaging *thin* biological samples with thicknesses of only a few tens of micrometers. Complex biological samples (e.g., multicellular complexes, such as organoids [14], tissue explants [15], embryos [16], etc.) are often thicker and exhibit *multiple scattering* . 3D RI reconstruction in such samples requires computational reconstruction frameworks that move beyond weak-scattering approximations and thus often employ nonlinear, iterative strategies. Recent examples include beam propagation-based methods [17,18], modified-Born series [19,20], transmission matrix approaches [21,22], physics-informed neural networks [23,24], and Lippmann–Schwinger-based formulations [25,26]. Using such frameworks, impressive imaging results have been obtained in various classes of multicellular scattering samples, such as organoids [14,27] and small organisms (e.g., *C. elegans*, tardigrades, mice, and zebrafish embryos) [28–30].

Notably, most inverse-scattering methods neglect absorption. Recent exceptions include works by Dong *et al.* [31] and Fan *et al.* [32]; however, these approaches either relied on weak-scattering assumptions or were demonstrated only in simulation using simplified, non-biological scattering samples. To the best of our knowledge, no method has experimentally unscrambled multiple-scattering effects in real-world biological samples that are *both* multiple scattering *and* absorptive, despite these being common characteristics of biological tissue. This leaves an important application gap since absorption often carries critical information about tissue composition, function, and physiological state that cannot be obtained from morphology alone. For example, absorbers endogenous to biological tissues, such as hemoglobin, melanin, lipids, and metabolic chromophores (e.g., cytochromes and flavins), provide insights into oxygenation, metabolism, and vascular density, which are not easily captured by the RI alone [33–35]. Other important, more application-specific examples include retinal tissues, which contain photoreceptors with well-characterized wavelength-dependent absorption. Obtaining absorption information from these tissues can provide insight into the visual transduction processes [36,37]. Plant tissues also contain strongly absorptive components, such as chlorophyll, carotenoid, and anthocyanin pigments [38,39]. Measuring their absorption can reveal biochemical traits (e.g., nitrogen content, specific leaf area, and water status) [40–43] and even physiological status, such as nutrient deficiency or senescence [43–45]. Reconstructing 3D absorption in multiple-scattering samples thus extends quantitative, label-free imaging to many biological and chemical systems where absorption carries functional information.

Importantly, though absorption and the RI are often treated as separate properties, they are fundamentally linked. Specifically, absorptivity is determined by the imaginary component of the complex-valued RI. Thus, inverse-scattering methods that reconstruct complex-valued RI should, in principle, inherently also recover the sample’s 3D absorptivity. To the best of our knowledge, this has not yet been demonstrated experimentally in multiple-scattering samples.

In this work, we demonstrate for the first time an inverse-scattering methodology that reconstructs 3D complex-valued RI in multicellular and heterogeneous biological samples after explicitly accounting for multiple-scattering effects. To accomplish this, we build upon our previous work with multi-slice beam propagation (MSBP), which employed a nonlinear physics-based model to reconstruct the *real-valued* RI from scattering measurements captured under various illumination angles [18,30]. In this work, we show that reconstructing the complex-valued RI with only angled-illumination measurements makes the inverse problem ill-posed. To address this challenge, we introduced optical refocusing as an additional source of measurement diversity. We validate that MSBP achieves correct convergence with this approach by reconstructing 3D absorptivity and the RI in various calibrated, dyed microspheres. We also conduct demonstrations in various multiple-scattering bio-samples containing endogenous absorbers. To the best of our knowledge, this work presents the first joint reconstructions of the 3D RI and absorptivity in samples thicker than 140 μm, exhibiting *both* multiple scattering *and* absorption. This represents an important step toward label-free volumetric imaging of scattering and absorptive biological tissues.

## 2. COMPUTATIONAL FRAMEWORK

### A. Multi-Slice Beam Propagation for Complex-Valued RI Reconstruction

We begin by introducing our inverse-scattering framework to reconstruct 3D absorptivity (jointly alongside the conventional 3D RI). As described above, this is achieved by extending the formulation for conventional MSBP toward reconstructing the *complex-valued* RI.

Conventional MSBP decomposes a 3D scattering sample into a sequence of infinitesimally thin layers, each represented by a 2D map of the *real-valued* RI [17,18]. The transmittance function corresponding to each layer thus acts to *phase-only* modulate the optical field incident to that layer. The sample’s overall scattering characteristics are approximated by accumulating the diffraction effects that occur as light propagates layer by layer through the sample.

Our extension to this model keeps a similar intuition, with the key difference that the RI is now allowed to be complex- valued. The real component corresponds to the conventional RI term, while the imaginary term encodes absorptivity. As a result, the transmittance function of each layer can be decomposed into a phase-only modulation term, as before, and an additional real-valued exponential-decay term, which accounts for absorption. Thus, as light propagates layer by layer through the sample’s complex-valued RI, it undergoes both phase modulation and absorption (see Fig. 1). We describe this mathematically.

Consider a 3D scattering sample decomposed into *M* layers separated by a distance Δz. In MSBP, light propagation through the sample is modeled as an alternating sequence of free-space propagation and layer-wise modulation steps. We mathematically describe this process below with vector/matrix variables, keeping a similar notation as that introduced by Kamilov *et al*. [17]:

(1) $$E_{m}(x)=diag\left(T_{m}(x)\right)\cdot H_{\Delta z}\cdot E_{m-1}(x).$$

Here, x=n+jk is a vector representing the sample’s 3D complex-valued RI. **n** and **k** are the real and imaginary components of **x**, representing the (conventional) RI and absorptivity, respectively. $E_{m-1}$ represents the field exiting the (*m* – 1)th layer. We matrix-multiply this field with the matrix operator $H_{\Delta z}$, which represents optically propagating $E_{m-1}$ a distance Δz to the *m*th layer. It is then spatially modulated by the *m*th layer’s transmittance function, $T_{m}(x)=exp\left(j\cdot \frac{2\pi }{\lambda }\cdot \Delta z\cdot \left(x_{m}-n_{0}\right)\right)$, where $n_{0}$ denotes the homogeneous, real-valued RI of the surrounding, non-absorptive media. $x_{m}=n_{m}+jk_{m}$ represents the sample’s *m*th layer complex-valued RI.

Modulation of the field propagated to the *m*th layer (i.e., $H_{\Delta z}\cdot E_{m-1}$) by that layer’s transmittance function corresponds to a spatially point-wise multiplication. This is represented in Eq. (1) as a matrix multiplication by the diagonal matrix diag(**T**_*m*_). The resulting field, denoted as $E_{m}$, represents the optical field exiting the *m*th sample layer. Starting from the illumination field incident to the sample, $E_{0}$, Eq. (1) repeated layer by layer, yielding the final field exiting the sample, $E_{M}$.

Notably, by decomposing $x_{m}$ into its real and imaginary components, $T_{m}$ can be re-expressed to explicitly capture its phase modulation and absorptive effects:

(2) $$T_{m}(x)=\text{exp}\left(j\cdot \frac{2\pi }{\lambda }\cdot \text{Δ}z\cdot \left(x_{m}-n_{0}\right)\right)\;\;=e^{-(2\pi /\lambda )\cdot \text{Δ}z\cdot k_{m}}\cdot e^{j(2\pi /\lambda )\cdot \text{Δ}z\cdot \left(n_{m}-n_{0}\right)}.$$

From the expanded form of Eq. (2), we see that $T_{m}$ comprises a purely real-valued exponential term accounting for absorption (first term) and the conventional phase-only modulation term (second term). Thus, Eq. (2) describes how light is both phase-modulated and absorbed at each sample layer as it propagates through the volume. Notably, in the case where there is no absorption (i.e., k=0), Eqs. (1) and (2) naturally simplify to the conventional MSBP model [17,18].

In practice, measurements are acquired with the camera focused on a plane within the 3D sample volume. Thus, the MSBP *forward model* 𝒢 describing the field at the measurement plane can be written as

(3) $$𝒢(x)=H_{\Delta \overset{ˆ}{z}}\cdot E_{M}(x).$$

Recall that $E_{M}$ denotes the field exiting the final (i.e., *M*th) sample layer. Matrix multiplication by $H_{\Delta \overset{ˆ}{z}}$ corresponds to back-propagating this field by a distance $\Delta \overset{ˆ}{z}$ to the in-focus imaging plane within the sample. Notably, the optical propagation operator **H** in Eqs. (1) and (3) was implemented in practice using the angular spectrum method in the spatial-frequency domain while also simultaneously applying spatial-frequency filtering to enforce a diffraction-limited pupil function [46].

### B. Inverse Problem

Thus, for a given volumetric RI distribution **x**, the forward model 𝒢(x) in Eq. (3) predicts the scattered field at the measurement plane. In practice, our goal is to recover **x** from the measurements, which constitutes the *inverse problem*. Because RI is a three-dimensional quantity while each measurement is inherently two-dimensional, this inverse problem is fundamentally ill-posed. Robust reconstruction therefore requires acquiring a sequence of measurements with sufficient diversity to adequately constrain the inverse problem.

To accomplish this, we acquired multiple scattering measurements of the sample being illuminated with various angled plane waves as well as being defocused to multiple positions. Consider the sample illuminated with the ℓ th plane wave and defocused to the *p*th position. In this case, we can generalize the MSBP forward model defined in Eq. (3) as ${𝒢}^{\left(\ell ,p\right)}(x)$, where ℓ and *p* index the illumination angle and the sample-defocus distance, respectively. Notably, the *p*th sample-defocus distance can be naturally incorporated by generalizing $\Delta \overset{ˆ}{z}$ in Eq. (3) as $\Delta {\overset{ˆ}{z}}^{\left(p\right)}$.

The inverse-scattering framework for reconstructing the sample’s complex-valued RI follows naturally. We formulate this problem as a least-squares optimization that searches for the 3D RI distribution that minimizes the discrepancy across all measurements between the measured scattering *amplitudes* (i.e., square root of raw intensity) and the *amplitude* predictions made by the MSBP forward model in Eq. (3):

(4) $$\hat{x}=\underset{x}{\text{arg min}}\frac{1}{LP}\overset{P}{\underset{p=1}{\sum }}\overset{L}{\underset{\ell =1}{\sum }}{\left\|y_{\text{amp}}^{\left(\ell ,p\right)}-\left|{𝒢}^{\left(\ell ,p\right)}(x)\right|\right\|}_{\text{L}2}^{2}+\alpha \cdot \text{TV}(x).$$

Here, $y_{\text{amp}}^{\left(\ell ,p\right)}$ denotes the measured scattering *amplitude* corresponding to the ℓ th plane-wave illumination and the *p*th sample-defocus position. *L* and *P* denote the total number of illumination angles and sample-defocus positions, respectively. $\|\ldots {\|}_{L2}$ denotes the L2-norm operator. The term TV(…) denotes the 3D total-variation regularizer, which stabilizes the iterative optimization process from noise and poor conditioning while also mitigating missing-cone artifacts [47]. *α* sets the strength of regularization. In practice, TV regularization is implemented via a proximal operator; details are provided in Supplement 1, Section 1.

The inverse problem formulated in Eq. (4) is structurally similar to those presented in prior works [17,18,30]. A key distinction, however, lies in its implementation. In particular, solving Eq. (4) requires computing the gradient of the least-squares objective, which in previous formulations was derived under the assumption that the sample RI **x** was purely real-valued. In this work, **x** is explicitly complex-valued, and the least-squares term in Eq. (4) involves a field amplitude that contains explicit dependence on the complex conjugate of **x**. As a result, Eq. (4) defines a complex-valued, non-holomorphic optimization problem. Accordingly, we re-derived the gradient of the least-squares term using the Wirtinger calculus; full derivations are provided in Supplement 1, Section 1.

As we show below, our empirical results demonstrate that reconstructing the complex-valued RI from angle-varying illumination alone leads to an ill-posed inverse problem, producing RI reconstructions with pronounced artifacts. For this reason, we increased measurement diversity by combining angular illumination with sample defocus. We observed that this strategy provided additional constraints that enabled the accurate reconstruction of the sample’s complex-valued 3D RI. Details of the computational hardware and reconstruction times for different imaging volumes are provided in Supplement 1, Section 2.

## 3. EXPERIMENTAL METHODS

### A. Optical Design

We show in Fig. 2 the schematic of our optical system, implemented as an off-axis interferometric setup with angle-scanning illumination. Coherent illumination was provided by three laser diodes operating at blue, green, and red wavelengths (Thorlabs, CPS450, λ = 450 nm; Thorlabs, CPS532, λ = 532 nm; and Thorlabs, CPS635, λ = 635 nm). The diodes were used independently, with each source fiber-coupled into the system one at a time. A pair of lenses then expanded and collimated the output beam to create a plane wave with a Gaussian intensity profile.

A 50/50 beam splitter (BS) (Thorlabs, CCM1-BS013) split the incoming light into sample and reference beams that propagate through two separate arms of the system. In the sample arm, light traveled to a 2D scanning galvanometer (GM) (Thorlabs, GVS212), which steered the beam to illuminate the sample across a range of incident angles. The GM and the camera (ToupTek, IUA20000KMA, pixel pitch = 2.4 μm) were in conjugate imaging planes. From the GM, the beam was illuminated into the sample volume by a 4f system consisting of a tube lens (Thorlabs, ACT508-200-A-ML, f = 200 mm) and an objective lens (OBJ). The resulting scattered light from the sample was then imaged with another objective and tube lens to the camera. For characterization experiments with microspheres, we used identical high-resolution objective lenses for both illumination and imaging (Nikon 60×, 1.42 NA oil) to maximize resolution. For imaging thicker biological samples, we used objectives with a longer working distance (illumination: Olympus, 20×, 1.0 NA water; imaging: Nikon, 16×, 0.8 NA water).

The reference beam was directed by a mirror (M) onto the camera at an off-axis angle relative to the sample beam, creating interference fringes at the camera’s imaging plane. From this interferogram, standard principles from off-axis holography were used to measure the complex-valued field at the camera plane [48]. The position and tilt of the reference beam were aligned so that the reference and sample beams spatially overlapped, while in Fourier space the central DC component was sufficiently separated from the +/− first-order components, which contained the relevant complex-valued information of the sample field.

### B. Data Acquisition

Interferometric measurements were acquired as the sample was sequentially illuminated with multiple angled plane waves. Illumination angles were arranged along a spiral trajectory spanning the full extent of the imaging objective lens’s pupil area. Acquisition parameters are described for each acquired dataset in Supplement 1, Section 3.

From each interferometric measurement, the complex-valued field (amplitude and phase) was recovered by computationally isolating the first-order diffraction term of the interferogram in the spatial-frequency domain [48]. This process was repeated after translating the sample out of the field of view in order to collect background field measurements. The sample fields were then normalized with the corresponding background fields via complex-valued division. Finally, the amplitude-only “measurements” used as **y** in Eq. (4) above were synthetically generated by simply taking the magnitude of the normalized field measurements. Notably, the illumination angles for each measurement were computationally recovered with high precision directly from the non-normalized background field measurements by locating the DC peak in the spatial-frequency domain.

Lateral and axial resolutions in angle-scanning RI tomography are given by $d_{xy}=\lambda /2NA$ and $d_{z}=\lambda /\left(n_{0}-\sqrt{{n_{0}}^{2}-{NA}^{2}}\right)$, respectively, where we recall that *n*_0_ denotes the RI of the surrounding medium [49]. For the microsphere characterization experiments, a 1.42 NA oil-immersion (*n*_0_ = 1.515) objective was used, yielding lateral resolutions of *d_xy_*=160, 190, and 220 nm, and axial resolutions of $d_{z}=450$, 540, and 640 nm, for wavelengths λ = 450, 532, and 635 nm, respectively. For the biological imaging experiments, a 0.8 NA water-dipping objective ($n_{0}=1.33$ ) was used at a single wavelength of λ = 635 nm, giving a lateral resolution of $d_{xy}=400$ nm and an axial resolution of $d_{z}=2.4$ μm.

### C. Digital Defocusing to Increase Measurement Diversity

As described earlier, solving Eq. (4) involves navigating a non-holomorphic, complex-valued solution space. This space is generally highly nonconvex, which poses significant challenges for accurate convergence. Our experimental results (discussed below) indicated that angled illuminations alone were not sufficient to achieve accurate convergence for complex-valued RI reconstruction. Thus, we increased measurement diversity by also incorporating sample defocusing.

Because we collect complex-valued *field* measurements $\left(y_{\text{field}}\right)$, we could implement sample defocusing *fully computationally* by numerically propagating the measured field across varying distances, i.e., $H_{\Delta \overset{ˆ}{z}\prime }$· $y_{\text{field}}$, where $\Delta \overset{ˆ}{z}\prime$ corresponded to the defocus distance. This allowed us to defocus the sample *without physical sample movement or additional acquisition time*. The amplitude component of each digitally defocused field was then used as a “measurement” $y_{\text{amp}}$ within the inverse problem in Eq. (4).

In summary, we captured scattering field measurements for each sample at *L* illumination angles. Each angular field measurement was then numerically propagated across *P* defocus planes, after which the amplitude component of the field was extracted. Thus, each unique combination of angle and defocus can be represented as $y_{\text{amp}}^{\left(\ell ,p\right)}$ in Eq. (4), where ℓ and *p* index the illumination angles and defocus planes, respectively. Information of *L* and *P* for each sample, as well as specific sample-defocus positions, is provided in Supplement 1, Section 3. Importantly, $y_{\text{amp}}^{\left(\ell ,p\right)}$ and ${𝒢}^{\left(\ell ,p\right)}(x)$ in Eq. (4) both index the same physical sample-defocus position.

A key insight of this approach is that defocusing each angular measurement indirectly encodes sample-specific phase information despite each individual measurement term **y** in the solver being purely a real-valued amplitude. *We intentionally omit direct phase information within the inverse solver*; we have recently shown that in multiple-scattering samples, even real-valued RI reconstructions derived directly from complex-valued fields are prone to severe artifacts [30] (see Supplement 1, Section 4).

## 4. RESULTS

### A. Quantitative Characterization

To validate the quantitative imaging capabilities of our system, we first reconstruct the complex-valued RI of absorptive polystyrene microspheres (PolySciences, PolyBead polystyrene 10 μm diameter microspheres; blue: 18,138, yellow: 18,337, black: 24,294, and violet: 18,139) immersed in non-absorptive, index-matching oil (Cargille Series A 1.572) and compare them with the microspheres’ “ground-truth” RI values. Importantly, while the RI of *undyed* polystyrene is well characterized across a broad wavelength range, these reference curves assume a purely real-valued RI and do not account for absorption, which is known to modify the real component of the RI [50].

Instead, to obtain ground-truth complex-valued RI values for the absorptive microspheres, we analyzed two-dimensional phase and amplitude maps extracted from field measurements acquired at the normal incidence of the illumination beam. Because a monolayer of microspheres well index-matched to the surrounding medium is weakly scattering, the measured 2D amplitude and phase maps corresponded to projections of the imaginary (absorptivity) and real (conventional RI) components of the microsphere’s complex-valued RI, respectively. Since the microspheres were homogeneous, say with a constant complex-valued RI of n+jk, we can write an expression for the measured complex-valued sample field:

(5) $$E_{\text{meas}}(\vec{r})=E_{0}(\vec{r})\text{exp}\left(j\frac{2\pi }{\lambda }(\text{Δ}n+jk)L(\vec{r})\right)\;\;=E_{0}(\vec{r})e^{-(2\pi /\lambda )kL(\vec{r})}e^{j(2\pi /\lambda )\text{Δ}nL(\vec{r})}.$$

Here, $E_{\text{meas}}$ and $E_{0}$ denote the measured and incident complex-valued fields, respectively, expressed as functions of the two-dimensional *vector* of spatial coordinate $\vec{r}=(x,y)$. Here, *x* and *y* are *scalar* variables representing the two orthogonal Cartesian directions. The quantity $\Delta n=n-n_{0}$ represents the RI contrast between the microsphere’s real-valued RI component (*n*) and that of the surrounding medium $\left(n_{0}\right)$. The term $L(\vec{r})$ denotes the physical path length through the sample at position $\vec{r}$ . For a microsphere, $L(\vec{r})$ takes the form of a half-ellipse, with an extent and center peak both equal to the microsphere’s physical diameter.

To isolate the microsphere-specific contribution to the field, we normalized the measured field by the incident field, $E_{\text{sample}}=E_{\text{meas}}/E_{0}$. From this normalized field, the imaginary (i.e., absorptivity, *k*) and real (i.e., conventional RI, *n*) components of the microsphere’s complex-valued RI could be directly inferred from the amplitude (i.e., $\left|E_{\text{sample}}\right|$) and phase (i.e., $∠E_{\text{sample}}$), respectively:

(6) $$k=-\frac{\lambda }{2\pi L(\vec{r})}\text{ln}\left(\left|E_{\text{sample}}(\vec{r})\right|\right),$$

(7) $$n=\frac{\lambda }{2\pi L(\vec{r})}∠E_{\text{sample}}(\vec{r})+n_{0}.$$

Using these relationships, we computed “ground-truth” values for *n* and*k* that provided the best fit to the measured amplitude and phase distributions across the centers of the microspheres. Figure 3 shows a representative fit to the measured phase and amplitude profiles of a blue-dyed microsphere with a diameter of 8.54 μm, recorded using red illumination (λ = 635 nm). We repeat this process for all colored microspheres across blue, green, and red illuminations. Ground-truth values for absorptivity (i.e., *k*) and RI contrast (i.e., Δn) are provided in Table S5.1 of Supplement 1, Section 5. We report $\Delta n=n-n_{0}$ rather than *n* because both *n* and $n_{0}$ depend on the wavelength, and reporting Δ*n* provides a more concise representation in Table S5.1 of Supplement 1.

Next, we reconstructed the 3D RI of the dyed microspheres by solving the inverse problem given in Eq. (4). Figure 4 shows the reconstructed 3D RI contrast for the same blue microsphere as before using: (1) the traditional MSBP formulation, which assumes a real-valued RI and uses the corresponding gradient and (2) our extended MSBP framework for complex-valued RI reconstruction, which employs the complex-valued gradient derived in Supplement 1, Section 1.

When the inverse problem was solved using the gradient derived for real-valued RI reconstruction, the resulting RI distribution was severely inaccurate and exhibited sharp discontinuous spikes of RI values surrounded by regions of extremely low RI. Figure 4(a) shows this failed reconstruction specifically in the case when only angular scattering measurements were used—however, similarly failed results were obtained even in the case when digitally defocused measurements were included (see Supplement 1, Section 6). This outcome was expected as the assumption of a purely real RI was fundamentally inconsistent with absorptive microspheres.

In contrast, using the gradient derived for complex-valued RI reconstruction led to immediate and substantial improvements. As shown in Figs. 4(b) and 4(e), reconstructions using only angular scattering measurements revealed a clear silhouette of the microsphere in both RI and absorptivity and were qualitatively more representative of the ground truth than the reconstruction in Fig. 4(a). However, line profiles through the center of the volume indicated that quantitative accuracy remained poor for the reconstructed RI. In contrast, when the reconstruction was repeated using *both* angular and digitally defocused measurements [Figs. 4(c) and 4(f)], both the 3D reconstructed RI and absorptivity were of high quality and high quantitative accuracy.

Results shown in Fig. 4 correspond to a blue-dyed microsphere illuminated with red light (λ=635nm), for which the sample exhibited the strongest absorption. Line-profile analysis yielded RI and absorptivity errors of $n_{\epsilon }<9.3\times 10^{-4}$ and $k_{\epsilon }<1.9\times 10^{-3}$, respectively, competitive with accuracies reported by other RI imaging methods [5,51]. We repeated this analysis across all dyed microspheres and illumination wavelengths and summarized the values in Table S5.1 of Supplement 1, Section 5. Notably, the reconstruction accuracy was observed to be inversely correlated with sample absorptivity. We speculate that this was due to the reduced signal-to-noise ratio (SNR) associated with higher absorption. Even after adjusting the regularization strength *α* in Eq. (4) to compensate for the SNR, reconstruction accuracy still showed modest degradation with higher absorption. For example, a yellow-dyed microsphere was substantially less absorptive under red illumination and achieved $n_{\epsilon }<1\times 10^{-5}$ and $k_{\epsilon }<1\times 10^{-5}$, more than an order of magnitude *lower* than the errors obtained for the blue-dyed microsphere under the same illumination conditions.

Lastly, we verified that this complex-valued MSBP formulation also remains applicable to non-absorptive samples, where it produced accurate RI reconstructions comparable to those obtained with the traditional real-valued MSBP formulation (see Supplement 1, Section 7). Importantly, this indicates that the proposed framework can generalize to *both* absorptive and non-absorptive samples in practice.

### B. Biological Weak-Scattering Sample

We next apply our technique to a moderately scattering yet strongly absorptive biological sample. Specifically, we image *Micrasterias radiata* (MR) algae, which exhibit strong chlorophyll-related absorption in the red and blue regions of the visible spectrum. This is evident in Fig. 5(a), which shows a bright-field image of an alga acquired under red light, revealing extensive absorptive regions throughout the specimen. We use our MSBP inverse-scattering framework to reconstruct this sample’s 3D complex-valued RI.

Figure 5(b) shows reconstruction results obtained using the traditional implementation of MSBP, where only angular scattering measurements were used in the inverse solver and the gradient was derived under the assumption of the real-valued RI. As expected, because this assumption was strongly mismatched to the absorptive nature of the sample, pronounced reconstruction artifacts were observed. In particular, the reconstructed RI exhibited severe saturation artifacts, with values spiking to non-physiological levels, especially in regions known to exhibit strong absorption based on the widefield image.

We then reconstructed the 3D RI using the extended MSBP formulation presented in this work, where the gradient was derived for complex-valued RI reconstruction. Figures 5(c) and 5(g) show the reconstruction results when only angular-illumination measurements were used in the inverse solver. Similar to our observations with the microspheres, this approach yielded substantial improvements over real-only reconstructions—saturation artifacts were eliminated, and absorptivity information became visible. However, the reconstructed RI exhibited limited contrast, with many internal features being poorly resolved. This behavior mirrored the microsphere results, where the RI component was strongly underestimated when only angular measurements were used to solve the inverse problem.

Figures 5(d) and 5(h) show complex-valued RI reconstruction when *both* angular and digitally defocused measurements were used in the inverse solver. In this case, both the RI and absorptivity were reconstructed with high contrast and fidelity. The characteristic morphology of *Micrasterias radiata* was clearly resolved, such as its two mirror-image semicells joined at a narrow central isthmus, as well as the subdivision of each semicell into multiple lobes arranged in a star-like pattern. Prominent chloroplasts within each semicell are clearly visible in the absorptivity maps, exhibiting a lobed architecture that follows the overall cell geometry. The strong, wavelength-dependent absorption is attributed to photosynthetic pigments (primarily chlorophylls and carotenoids [52]) concentrated within these chloroplasts. Each chloroplast contains numerous pyrenoids, recognizable by their circular starch sheaths in the RI contrast [53], which are known to be sites of carbon fixation [54]. The rigid cell wall was also resolved, revealing fine structural features such as marginal indentations and surface patterning [55–58]. Together, the reconstructions in Fig. 5 highlight the pronounced morphological organization and intracellular compartmentalization characteristic of this alga.

### C. Biological Multiple-Scattering Samples

#### 1. Succulent

We next applied our technique to biological specimens characterized by both scattering and absorption, beginning with a tissue section of a common succulent. We first confirmed that this specimen was multiple scattering by measuring an extinction mean-free path substantially smaller than the sample thickness (Supplement 1, Section 8) and by also demonstrating that the weak-scattering Rytov approximation produced poor 3D RI reconstruction results (Supplement 1, Section 9).

Figure 6 shows the MSBP-based inverse-scattering reconstruction of the sample’s 3D RI and absorptivity. The inverse problem was solved using the gradient for the complex-valued RI while leveraging measurement diversity from both angular and defocused scattering measurements. Much like the previously analyzed alga, succulents exhibited chlorophyll-related absorption, as demonstrated in the red-light bright-field image in Fig. 6(a). As indicated by the arrows, dark regions were primarily concentrated within the micron-scale chloroplasts as well as the parenchyma cell walls surrounding the internal water-filled vacuoles.

In Figs. 6(c1), 6(c2) and 6(d1), 6(d2), we show x–y and x–z maximum projections (computed across 50 adjacent planes) of the reconstructed RI and absorptivity volumes, respectively. The x–z projections in Figs. 6(c2) and 6(d2) indicate a tissue section thickness of approximately 145 μm. The absorptivity reconstruction prominently highlights the parenchyma cell walls and chloroplasts, consistent with their absorptive contrast observed in the bright-field image. Notably, these same structures also appear with high contrast in the RI maps, indicating that regions of elevated RI and absorptivity are largely spatially co-localized in this sample. Figures 6(e1), 6(f1) and 6(g1), 6(h1) show RI and absorptivity from single x–y cross-sectional planes at axial positions $z_{1}=43.6$ μm and $z_{2}=90.38$ μm, respectively. Magnified views of the dashed yellow regions [see Figs. 6(e2), 6(f2), 6(g2), and 6(h2)] reveal chloroplasts positioned along the inner periphery of the cell wall, consistent with the known cellular organization [59]. Figure 6(g2) additionally shows a nucleus located adjacent to the cell wall. As nuclei are predominantly phase shifting rather than absorptive structures [56], they exhibit strong RI contrast but minimal absorptive contrast, explaining the nucleus’s clear appearance in Fig. 6(g2) but virtually undetectable signature in Fig. 6(h2). Interestingly, even when using the gradient for the real-only RI, the reconstructed RI appeared reasonable and partially captured some key features, such as cell walls and chloroplasts. This was observed likely because the succulent tissue was only weakly absorptive, as reflected by the lower contrast in its bright-field image relative to that of the strongly absorptive algal sample [see Fig. 5(a) from before]. As a result, the mismatch introduced by neglecting absorption produced less severe reconstruction artifacts than in the algal case. Nevertheless, limitations remained. For example, chloroplasts were either reconstructed with reduced contrast [Fig. 6(e3)] or high-frequency saturation artifacts [Fig. 6(g3)]. Importantly, these artifacts were spatially correlated with regions of elevated absorptivity, mirroring the behavior observed in the algal reconstructions.

In this example, regions of elevated RI and absorptivity were largely spatially co-localized. In the next section, we reconstructed a sample in which regions of high RI and absorption are physically separate.

#### 2. Zebrafish Embryo (48 h Post-Fertilization)

Lastly, we demonstrate 3D RI reconstruction of the tail region of a 48 h post-fertilization (hpf) zebrafish embryo (sample preparation described in Supplement 1, Section 10), a widely used multicellular system that provides insight into embryogenesis and organ formation. However, imaging the zebrafish embryo at subcellular resolution is often challenging due to its complex architecture, which induces substantial multiple light scattering. As with the previous succulent specimen, we confirmed that this sample was indeed multiple scattering by measuring a mean-free path smaller than the sample thickness (Supplement 1, Section 8) and by showing that the weak-scattering Rytov approximation yielded poor 3D RI reconstructions (Supplement 1, Section 9).

In prior work, we applied MSBP to image a 24 hpf zebrafish embryo and successfully reconstructed rich structural detail using label-free RI contrast. At that stage, the embryo is largely non-absorptive, so assuming a real-valued RI was sufficient. In contrast, by 48 hpf, melanophore development introduces significant optical absorption. As a result, the real-only RI assumption is no longer valid, necessitating the reconstruction of complex-valued RI.

Figure 7 shows reconstruction results obtained using our proposed framework for complex-valued RI recovery using measurements combining angular illumination and sample defocus. To illustrate the global structure of the embryo, we first show x–y and x–z maximum-intensity projections of both the reconstructed absorptivity and RI volumes. Figures 7(a1) and 7(a2) show maximum projections of absorptivity in the x–y and x–z planes. Although the embryo remained largely transparent, sparse regions of strong absorption were evident that extended across the whole embryo. The zoomed-in view in Fig. 7(a3) shows that these high-absorptivity regions corresponded to melanophores, identifiable by their morphology and consistency with prior reports [60–62].

Figures 7(b1) and 7(b2) show maximum projections of the reconstructed RI volume. The overall morphology of a 48 hpf zebrafish tail was clearly resolved, including the cardiac vein, dorsal aorta, notochord, and yolk extension. The fin mesenchyme was also clearly visualized, within which the developing fin rays were readily distinguishable. Figure 7(b3) shows a zoomed-in view of the region indicated by the yellow dashed rectangle in Fig. 7(b1), revealing distinct red blood cells located within the dorsal aorta and cardinal vein. Interestingly, red blood cells in the dorsal aorta exhibited measurably higher RI than those in the cardinal vein. As the dorsal aorta and cardinal vein carry oxygenated and deoxygenated blood, respectively, this contrast is consistent with prior studies demonstrating that hemoglobin’s RI varies with the oxygenation state [63,64], suggesting that the observed RI difference may at least in part reflect differences in blood oxygenation between the two vessels. This offers exciting preliminary evidence that inverse scattering may be capable of distinguishing arterial from venous blood within scattering vasculature.

For more detailed visualization of specific anatomical features, we examined x–y cross-sections within the dashed white rectangle in Fig. 7(b1) at depths z=94.76 μm and z=119.48 μm. To achieve clear lateral visualization despite the natural axial curvature of the zebrafish tail, these lateral cross-sections were digitally flattened along the dashed yellow line in Fig. 7(b2).

Figure 7(c1) shows the x–y cross-section at z=94.76 μm along the embryonic midline. The notochord was prominently visible, characterized by large, vacuolated cells arranged as repeated rounded compartments along the anterior–posterior axis. Individual cell membranes were clearly visible. Dorsal to the notochord, differentiated spinal cord tissue was visible, exhibiting dark, rounded structures consistent with neurons and other neural cell types. The dorsal aorta, clearly visible and indicated by the red dashed line, are the principal vessels running along the ventral surface of the notochord, and groups of red blood cells were clearly localized within its lumen, as expected.

Figure 7(c2) shows a zoomed-in view of the posterior region indicated by the yellow dashed rectangle in Fig. 7(c1). The neural tube, the early developmental precursor to the spinal cord at this axial level, was clearly visible in this zoomed-in view and exhibited its characteristic honeycomb-like tessellated appearance arising from tightly packed, proliferating neuroepithelial progenitor cells. The neural tube lumen, a hollow fluid-filled cavity running along the length of the neural tube, is indicated by the blue dashed line, and the floor plate cells adjacent to the notochord are indicated by the green dashed line.

Figure 7(c3) shows the x–y cross-section at *z* = 119.48 μm. Distinct myotome bundles forming the segmented trunk musculature were clearly visible with their characteristic V-shaped architecture. Intersegmental vessels branching from the dorsal aorta and extending dorsally between adjacent myotomes were also resolved. Figure 7(c4) shows a zoomed-in view of the yellow dashed rectangle in Fig. 7(c3), revealing that individual muscle fibers within each myotome bundle can be resolved as elongated, parallel structures oriented along the anterior–posterior axis. Notably, clear striations are visible within individual fibers, arising from the organized sarcomeric architecture. This striated pattern, a defining feature of skeletal muscle, highlights the ability of our approach to resolve subcellular organization in a multicellular organism that is both scattering and absorptive.

## 5. DISCUSSION AND CONCLUSION

In this work, we introduced an inverse-scattering methodology to reconstruct the 3D RI and absorptivity in biological samples that exhibit both scattering and absorption. Building on the traditional MSBP framework, we extended the mathematical formulation to explicitly incorporate the *complex-valued* RI in the gradient update of the inverse solver. This allowed us to leverage the fact that absorptivity is intrinsically linked to the conventional RI as the imaginary component of the more generalized complex-valued RI. While other inverse-scattering approaches have previously demonstrated 3D RI reconstruction in scattering samples, absorption has largely been neglected, either in the reconstruction formulation or in the experimental demonstration. To the best of our knowledge, the results presented here constitute the first experimental demonstration of joint 3D reconstruction of both the RI and absorptivity in samples that are simultaneously scattering and absorptive.

Interestingly, reformulating the gradient alone was not sufficient to achieve high-quality complex-RI reconstruction in real-world samples. Solving for a complex-valued RI requires solving a highly nonconvex optimization problem over a complex-valued solution space, which is more underdetermined than the corresponding real-valued reconstruction problem. Thus, additional constraints on the search space were essential. In this work, we introduced such constraints by increasing measurement diversity. Specifically, we combined angular scattering with sample-defocused measurements within the MSBP inverse-scattering framework. Using this strategy, we demonstrated robust reconstruction of volumetric RI and absorptivity across a range of samples, including calibration microspheres and biological samples.

There are several promising directions for future research. For example, one limitation of MSBP is that it assumes *forward* light scattering and does not model *backscattered* light. For absorptive samples that are also sufficiently thick or heterogeneous, backscattering can become significant, causing a portion of the scattered light to never reach the detector. Because this signal is not modeled by the MSBP forward model, the inverse solver may incorrectly attribute this loss to absorption, potentially introducing reconstruction artifacts. Developing inverse-scattering methods to explicitly account for both absorption and backscattering as distinct forms for attenuation is an important direction for future work.

Another natural extension is toward hyperspectral tomography, where illuminating the sample at multiple wavelengths could enable the reconstruction of four-dimensional spatio-spectroscopic datasets capturing wavelength-dependent volumetric RI and absorptivity. Importantly, spectral dispersion and absorption provide complementary contrast mechanisms that connect tissue structure to composition and physiological state. Access to both could enable powerful label-free differentiation of biological samples that appear morphologically similar yet differ in biochemical makeup, metabolism, or functional activity.

Extending to hyperspectral imaging will also motivate replacing digital defocusing with physical defocusing. Most broadband illumination sources, such as LEDs and supercontinuum lasers, exhibit only partial coherence, making interferometric measurements either difficult or impractical. Thus, directly acquiring intensity images at multiple physical defocus planes may become a more practical means of introducing defocus diversity. An additional advantage is that physical defocusing avoids the numerical propagation artifacts associated with digital defocusing, such as aliasing at large propagation distances. However, achieving physical defocusing in practice will require addressing several implementation challenges, including the effects of partial coherence, depth-dependent aberrations, and varying noise characteristics across defocus planes.

Another exciting direction is to study the challenges introduced by the expanded solution space when reconstructing the complex-valued RI compared to the real-valued RI. Such an analysis could provide valuable insight into how measurement strategies should be designed to better constrain the inverse problem. For example, our recent companion work has shown that as samples become increasingly thick and scattering, combining angular illumination with defocused measurements is already essential for accurately reconstructing even just real-valued RI [30]. Reconstructing the complex-valued RI in similarly thick samples would further degrade the conditioning of the inverse problem, such that combining angular and defocus measurements may no longer provide sufficient constraints for accurate reconstruction. Thus, identifying strategies that provide further constraints will be critical. For example, one strategy could be to optimize the number and axial placement of defocus planes, so as to maximize the information diversity within the measurements and improve the conditioning of the inverse problem. Hyperspectral measurements, mentioned above, offer another promising strategy. Because the RI and absorptivity typically vary smoothly with wavelength, smoothly varying priors could potentially be leveraged to constrain the inverse problem when a spectral dataset is captured. Lastly, one could explore strategies to illuminate the sample with structured patterns that are richer and more complex than the angular illumination and sample defocus schemes explored here. Such patterned illuminations could offer greater flexibility in tailoring the measurement diversity to optimally constrain the inverse-scattering problem across different classes of scattering and absorptive samples.

Overall, this work represents an important advance in inverse-scattering methods. By explicitly accounting for absorption, our approach enables accurate reconstruction of both the RI and absorptivity in biological tissues, paving the way for more comprehensive, label-free volumetric imaging of biological samples that are both scattering and absorptive.

## Supplementary Material

supplementary

See Supplement 1 for supporting content.

## Figures and Tables

**Fig. 1.**
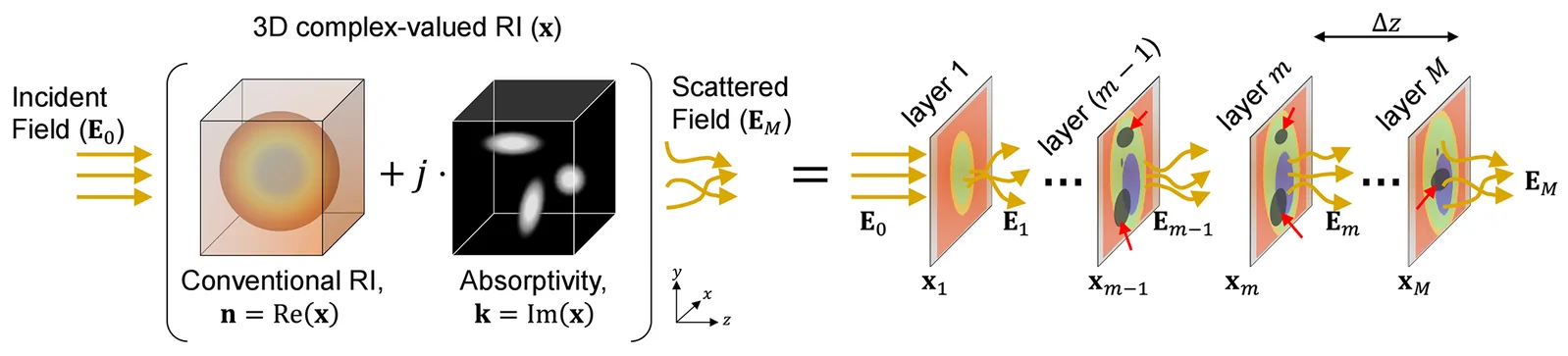
Illustration of the MSBP forward model for absorptive scattering samples. Samples are represented as 3D complex-valued RI volumes, comprised of terms for the conventional RI and absorptivity. In the layer-by-layer depiction, color represents the conventional RI, while dark regions (marked by red arrows) highlight areas of absorption.

**Fig. 2.**
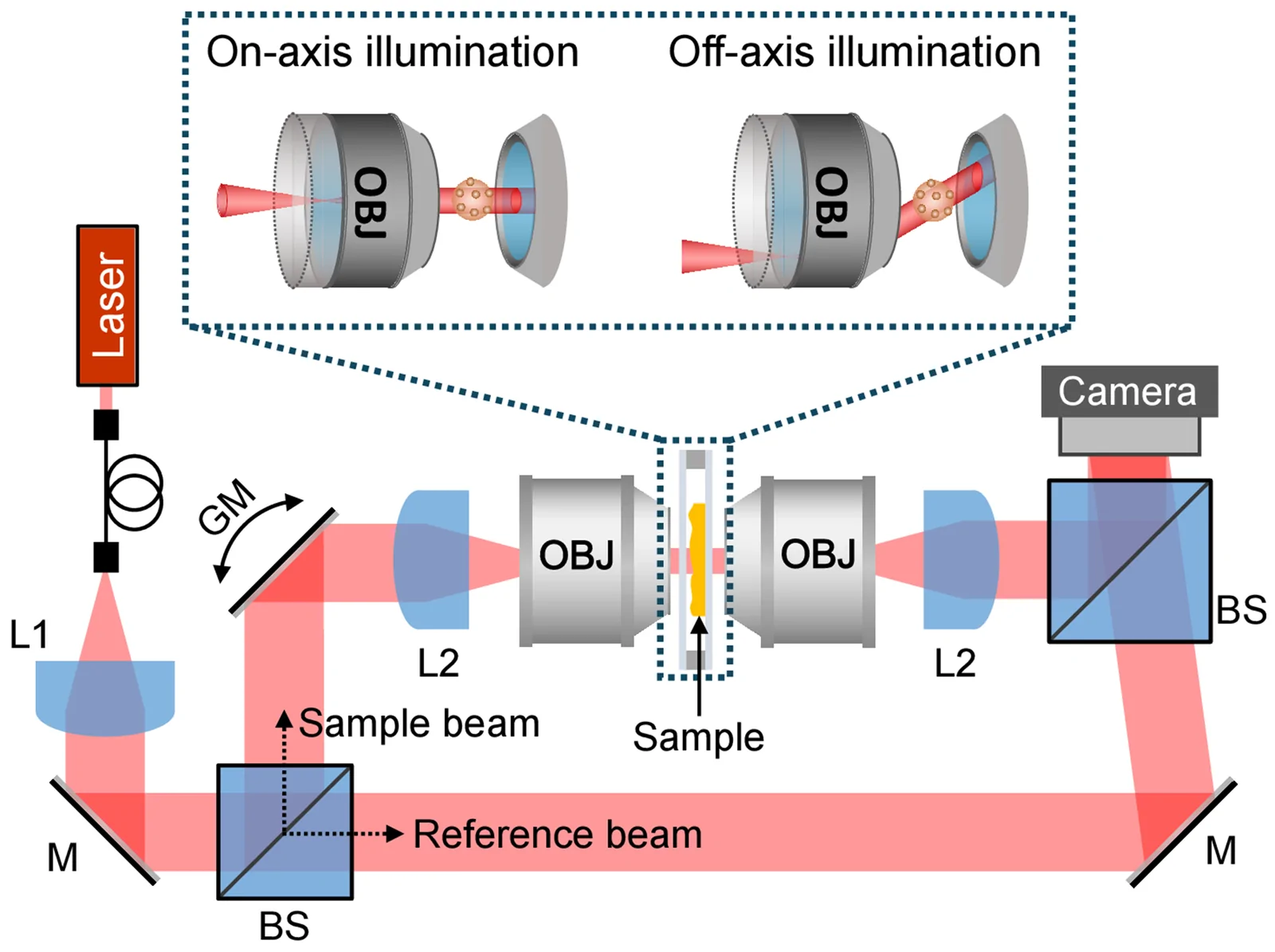
Optical schematic of an angle-scanning, off-axis interferometric imaging system. For illustration simplicity, only one laser source is shown. The first beam splitter (BS) splits light into sample and reference beams. The sample beam is directed to a galvo mirror (GM), which steers the illumination beam onto the sample, through 4f system L2 and OBJ. Scattered light from the sample is imaged through an identical 4f system onto the camera. The reference beam is directed to the camera at an off-axis angle from the sample beam. Reprinted from [30] under a Creative Commons license.

**Fig. 3.**
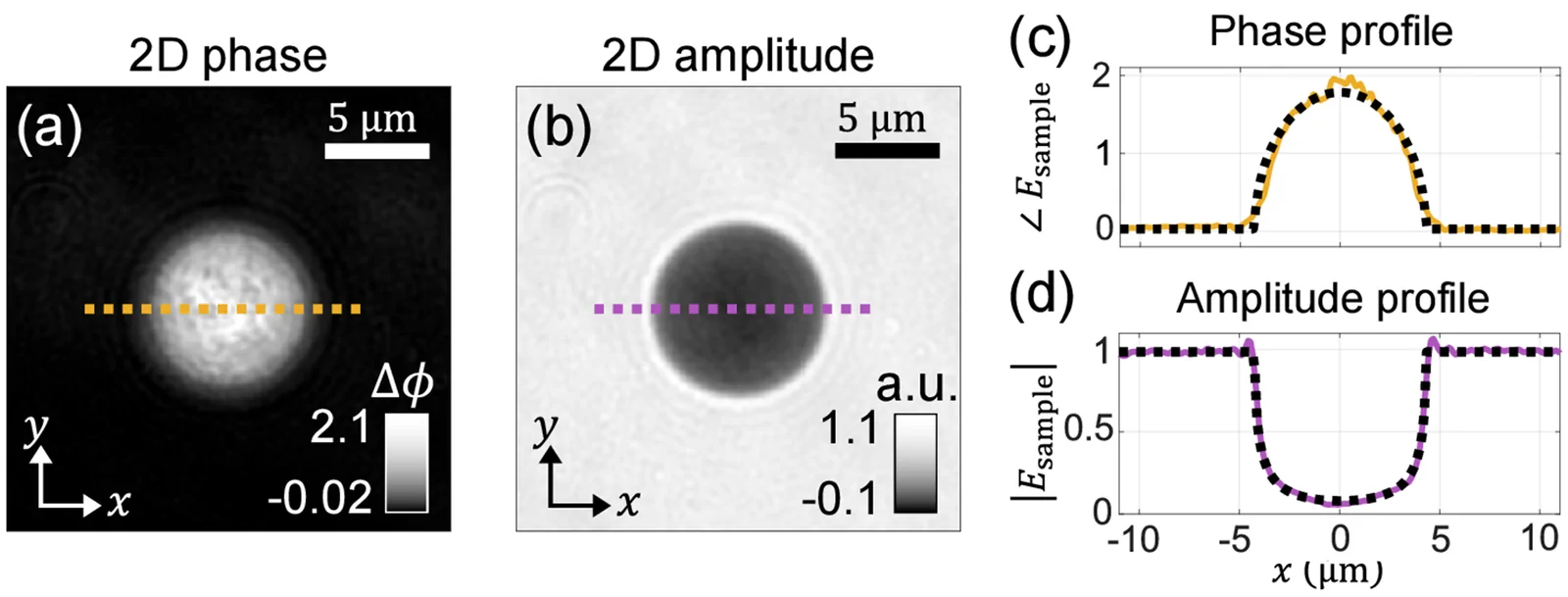
Ground-truth RI (*n*) and absorptivity (*k*) values of the calibration microspheres are obtained by fitting model profiles to those extracted from 2D (a) phase and (b) amplitude maps measured under orthogonal illumination. Line profiles taken along the dashed yellow and purple paths in (a) and (b) are shown as colored curves in (c) and (d), respectively. The black dashed curves show the fitted phase and amplitude profiles corresponding to the inferred ground-truth values of *n* and *k*.

**Fig. 4.**
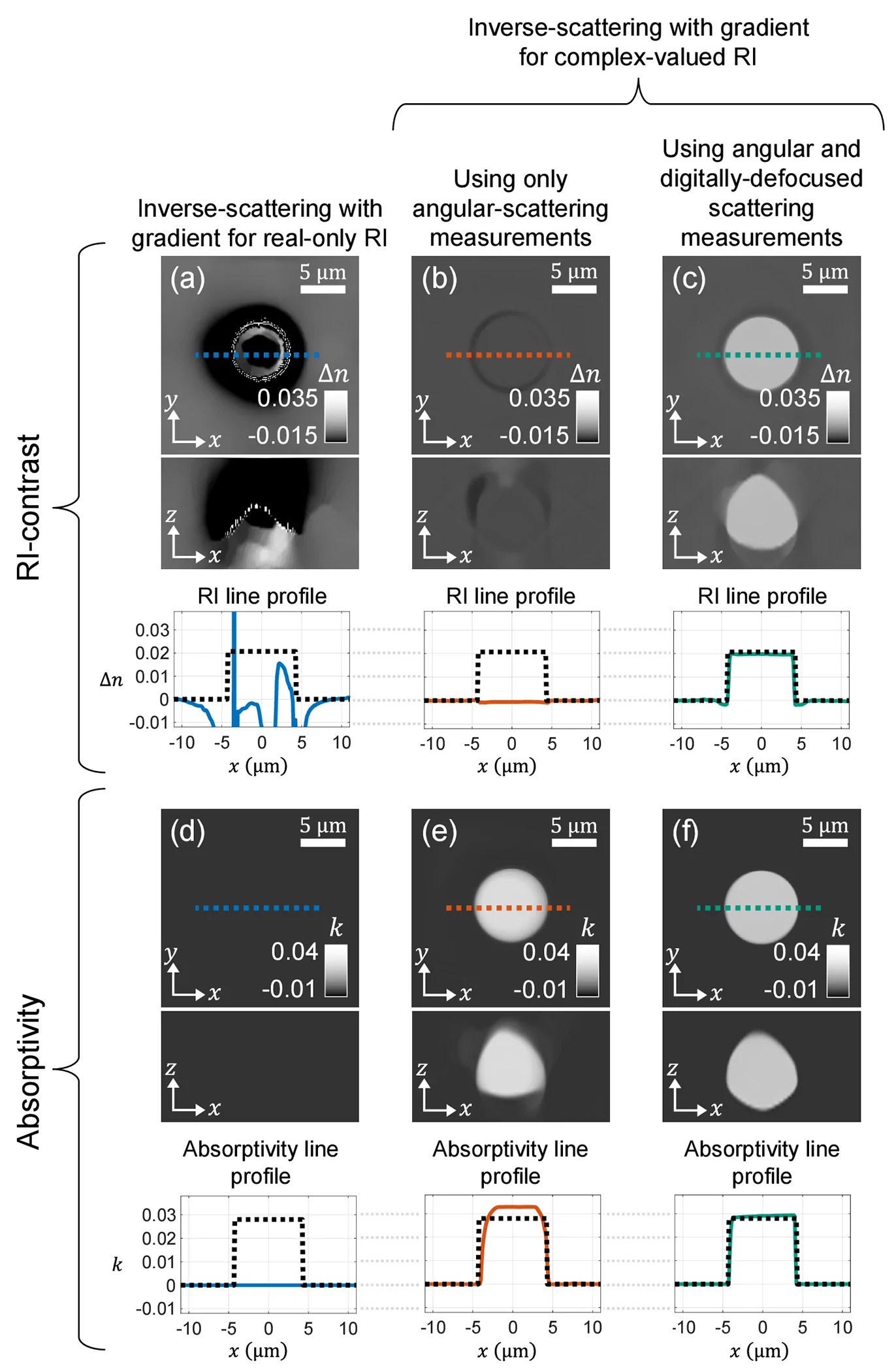
Comparison of the reconstructed 3D complex-valued RI (i.e., the conventional RI and absorptivity) of an absorptive (blue-dyed) microsphere. Reconstructions were obtained via MSBP-based inverse scattering with a (a) gradient formulated for the real-only RI and using data from angular scattering measurements, as well as a gradient formulated for the complex-valued RI and using (b) only angular scattering measurements and (c) both angular and defocused scattering measurements. Line profiles extracted along the dashed lines in (a)–(f) are shown to assess quantitative accuracy across all reconstruction scenarios. Black dashed curves in the plots denote the “ground-truth” RI and absorptivity values computed from measured 2D fields, as described previously in Fig. 2.

**Fig. 5.**
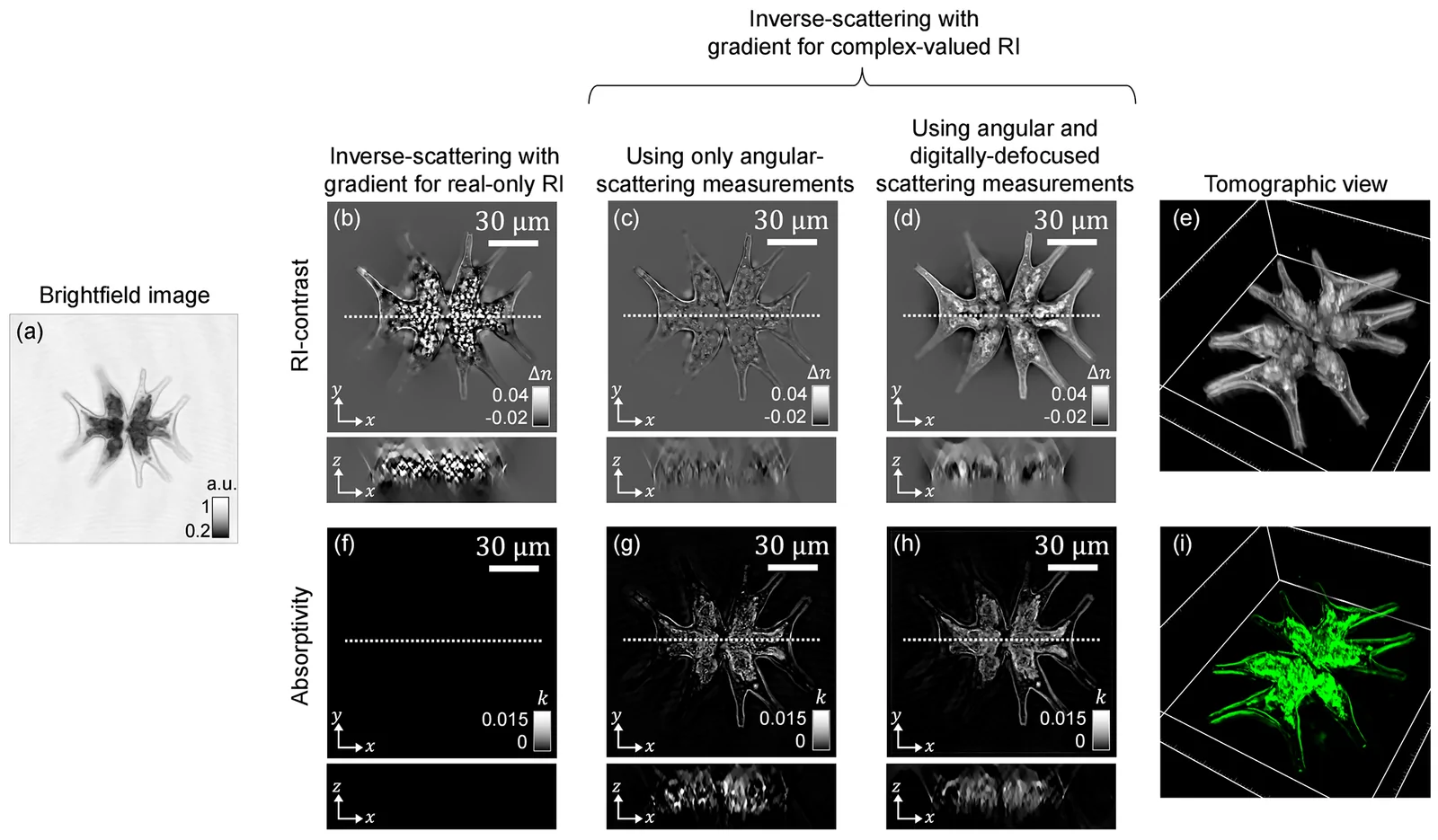
(a) Bright-field image of *Micrasterias radiata* under red illumination. Chlorophyll within the cell absorbs red light, producing darker regions corresponding to chlorophyll-rich structures. x–y and x–z cross-sections through the center of the reconstructed 3D RI are shown, where RI reconstructions are obtained using MSBP-based inverse scattering with (b) gradient for the real-only RI and using angular scattering measurements, (c) gradient for the complex-valued RI with angular scattering measurements, and (d) gradient for the complex-valued RI with combined angular *and* defocused scattering measurements. Panels (f)–(h) show the corresponding cross-sections of the reconstructed 3D absorptivity maps. (e) and (i) Tomographic views are shown of the reconstructed RI and absorptivity maps in (d) and (h), respectively. An animated version of this 3D rendering is provided in Visualization 1.

**Fig. 6.**
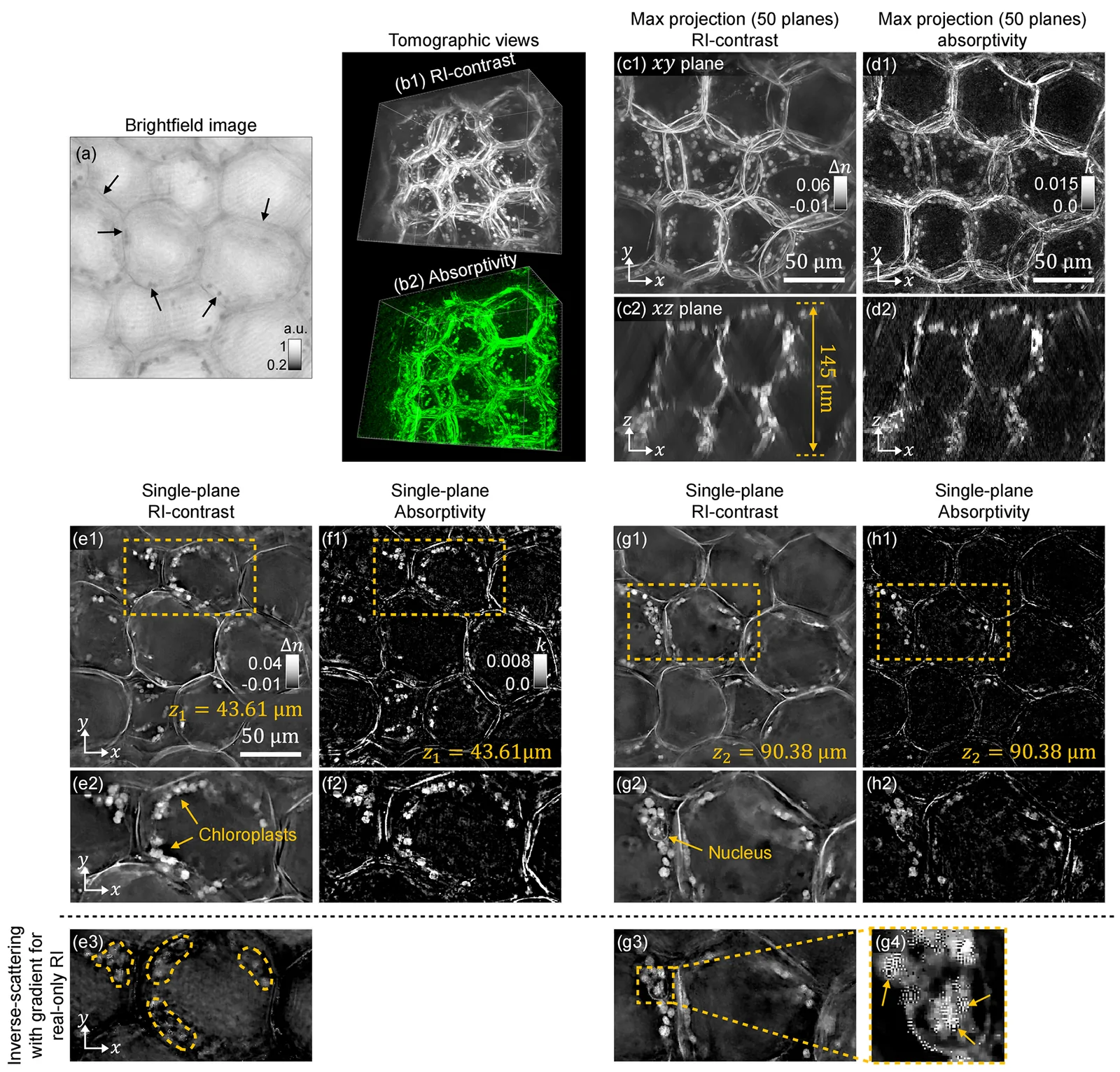
(a) Bright-field image of a 145 μm thick succulent tissue section. (b1) and (b2) Tomographic renderings of the reconstructed 3D RI and absorptivity volumes obtained via MSBP-based inverse scattering using a complex-valued RI gradient and incorporating both angular and defocused scattering measurements. (c1) and (c2) Maximum x–y and x–z projections (computed over 50 planes) of the RI volume are shown, as well as (d1) and (d2) the corresponding absorptivity projections. (e1), (f1) and (g1), (h1) show x–y cross-sections of the RI and absorptivity volumes at $z_{1}=43.61$ μm and $z_{2}=90.38$ μm, respectively. (e2), (f2), (g2), and (h2) Magnified views are shown of the dashed yellow regions in (e1), (f1), (g1), and (h1), respectively, highlighting chloroplasts and cell boundaries with RI and absorptivity contrast. (e3) and (g3) Notably, magnified views of the same regions reconstructed using a real-only RI gradient exhibit pronounced degradation, including reduced contrast [yellow dashed regions in (e3)] and high-frequency saturation artifacts [indicated by yellow arrows in (g4)]. See Visualization 2 for an animated version of this 3D rendering.

**Fig. 7.**
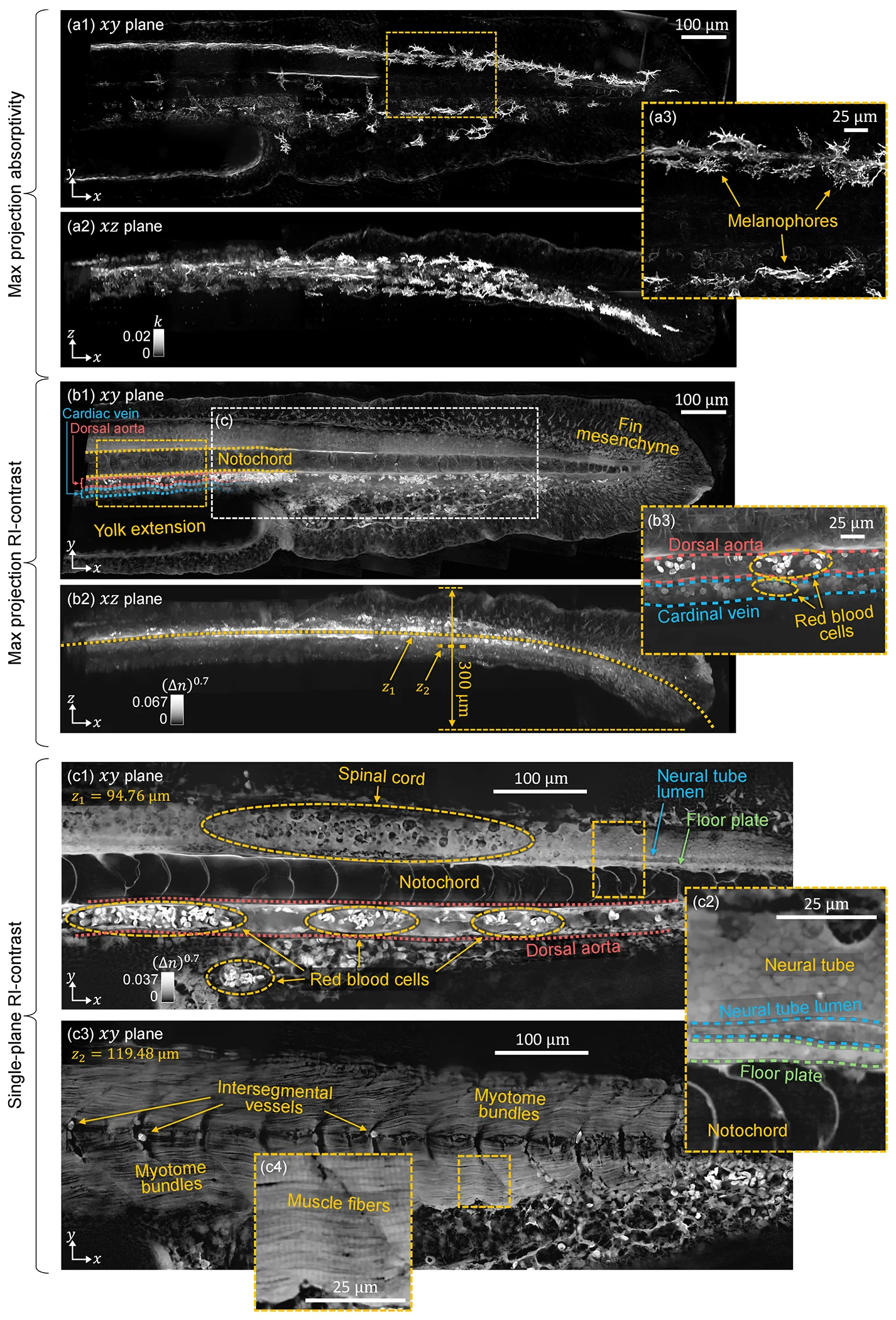
(a1) and (a2) Maximum-intensity projections of 48 hpf zebrafish embryo’s reconstructed absorptivity in the x–y and x–z planes, with the (a3) zoomed-in view identifying melanophores as the dominant absorptive feature. (b1) and (b2) Maximum-intensity projections of the embryo’s reconstructed RI in the x–y and x–z planes show the overall tail morphology, clearly visualizing the cardinal vein, dorsal aorta, and notochord. For visualization purposes, the RI images in (b1)–(b3) and (c1)–(c4) are displayed using power-law contrast scaling (exponent=0.7, indicated above the colorbar) to enhance the visibility of low-contrast structures. (b3) Zoomed-in view indicates the red blood cells visualized in the vasculature. (c1) Lateral cross-section at $z_{1}=94.76$ μm resolves the notochord, spinal cord, dorsal aorta, and red blood cells, as well as the (c2) the neural tube, neural tube lumen, and floor plate in the zoomed-in view. (c3) Lateral cross-section at $z_{2}=119.48$ μm reveals segmented myotome bundles and intersegmental vessels. (c4) Zoomed-in view shows the individual striated muscle fibers. See Visualization 3 for an animated version of this 3D rendering.

## Data Availability

The multi-angle biological tissue datasets used to generate the results in this work are available in Ref. [65]. The code used to perform the phase-retrieval reconstructions from these datasets is available in Ref. [66].
