# Supplementary material for: Inverse scattering of absorptive samples via beam propagation
Source: Optica. Author manuscript; Available in PMC 2026 Oct 15. (PMC13664592; doi:10.1364/optica.599869)
Supplement: Supplementary file 1 [file NIHMS2214116-supplement-supplementary.pdf]

Supplemental Document

OPTICA

Inverse scattering of absorptive samples via beam propagation: supplement

Peter Wagenaar,[1] Jeongsoo Kim,[1] Mary E. Swartz,[2] Johann K. Eberhart,[2] and Shwetadwip Chowdhury[1,*]

[1] *Department of Electrical and Computer Engineering, University of Texas at Austin, 2501 Speedway, Austin, Texas 78712, USA*
[2] *Department of Molecular Biosciences, University of Texas at Austin, 2501 Speedway, Austin, Texas 78712, USA*
[*] *shwetadwip.chowdhury@utexas.edu*

This supplement published with Optica Publishing Group on 11 September 2026 by The Authors under the terms of the Creative Commons Attribution 4.0 License in the format provided by the authors and unedited. Further distribution of this work must maintain attribution to the author(s) and the published article's title, journal citation, and DOI.

Supplement DOI: https://doi.org/10.6084/m9.figshare.33154094

Parent Article DOI: https://doi.org/10.1364/OPTICA.599869

Inverse-scattering of absorptive samples via beam propagation: supplemental document

S1. Gradient-based reconstruction of complex-valued refractive index

In this section, we derive the gradient of the loss function from Eq. (4) in the main text. This gradient is used within a gradient-descent framework to iteratively minimize the loss and recover a 3D complex-valued refractive index (RI) distribution of the sample. The derivation requires extending standard real-valued gradient methods to the complex domain. To accomplish this, we employ *Wirtinger calculus*, which provides a convenient framework for computing derivatives of real-valued loss functions with respect to complex variables. A brief overview of this framework is provided below following the treatment in [1].

1.1 Introduction to Wirtinger Calculus

Eq. (4) of the main manuscript defines a least-squares minimization over the L2 norm of the difference between experimentally measured data and simulated measurements produced by the forward model. When the refractive index is assumed to be *real-valued*, this optimization problem can be solved using conventional gradient-based reconstruction methods [2,3]. In the more general setting considered in this work, however, the refractive index is *complex-valued*. The real component represents the conventional refractive index contrast responsible for phase delay, while the imaginary component represents *optical absorption*. Incorporating this complex-valued representation introduces additional mathematical considerations when computing gradients. In classical complex analysis, derivatives of complex functions exist only if the function satisfies the *Cauchy–Riemann equations*, which implies that the function is *holomorphic*. The loss function used in this work involves the squared L2 norm of complex-valued quantities, which can be written as $\|\mathbf{a}\|_{L2}^2 = \mathbf{a}^H\mathbf{a}$. This expression maps a complex vector to a *real-valued scalar*. A fundamental result from complex analysis states that a *non-constant real-valued function of a complex variable cannot be holomorphic*, meaning that the conventional complex derivative is not defined. One possible approach to circumvent this issue is to rewrite the problem in terms of the *real and imaginary components* of the complex variable, thereby converting the optimization into a joint-variable real-valued problem. However, this approach typically leads to cumbersome expressions and obscures the natural structure of the complex-valued forward model. Instead, we adopt the *Wirtinger calculus framework*, which treats a complex variable and its complex conjugate as independent variables. A complex variable z and its conjugate are defined as:

$$z = x + j \cdot y, \qquad z^* = x - j \cdot y \tag{S1}$$

where x and y denote the real and imaginary components respectively. Within this framework, generalized derivatives for functions are defined with respect to *both* z and z^*:

$$\begin{aligned} \frac{\partial f}{\partial z} &= \frac{1}{2}\left(\frac{\partial f}{\partial x} - j\frac{\partial f}{\partial y}\right) \\ \frac{\partial f}{\partial z^*} &= \frac{1}{2}\left(\frac{\partial f}{\partial x} + j\frac{\partial f}{\partial y}\right) \end{aligned} \tag{S2}$$

Importantly, z and z^* are treated as *independent variables* in the Wirtinger framework (i.e., $\partial z/\partial z^* = \partial z^*/\partial z = 0$). In this case, f is treated as a function of both z and z^* (i.e., $f(z, z^*)$), enabling differentiation via Eq. (S2) *even when the function is not holomorphic*.

Importantly, this property extends naturally to multivariate variables where $\mathbf{z} \in \mathbb{C}^N$. Wirtinger calculus also preserves many familiar rules from standard calculus, including a generalized chain rule. For a composite function $f(g(\mathbf{z}, \mathbf{z}^*))$, the derivatives become:

$$\begin{aligned} \frac{\partial}{\partial \mathbf{z}} f\big(g(\mathbf{z}, \mathbf{z}^*)\big) &= \frac{\partial f}{\partial g} \cdot \frac{\partial g}{\partial \mathbf{z}} + \frac{\partial f}{\partial g^*} \cdot \frac{\partial g^*}{\partial \mathbf{z}} \\ \frac{\partial}{\partial \mathbf{z}^*} f\big(g(\mathbf{z}, \mathbf{z}^*)\big) &= \frac{\partial f}{\partial g} \cdot \frac{\partial g}{\partial \mathbf{z}^*} + \frac{\partial f}{\partial g^*} \cdot \frac{\partial g^*}{\partial \mathbf{z}^*} \end{aligned} \tag{S3}$$

This formulation enables gradient-based optimization directly in the *complex domain* without separating real and imaginary components. Importantly, the resulting gradient updates are mathematically equivalent to those obtained by optimizing over the real and imaginary variables separately, while yielding more compact expressions that better reflect the structure of complex-valued forward models. The notation introduced above will be used throughout the following derivations and further explanations of Wirtinger calculus can be found in [1,4].

1.2 Reconstruction methodology

3D RI reconstruction is obtained by minimizing an *amplitude-only* loss function that measures the *least-squares discrepancy between experimental measurements and corresponding predictions of the multi-slice beam propagation (MSBP) forward model*, aggregated across all measurements. This minimization was expressed in Eq. (4) in the main text:

$$\hat{\mathbf{x}} = \arg\min_{\mathbf{x}} \frac{1}{LP} \sum_{p=1}^{P} \sum_{\ell=1}^{L} \left\| \mathbf{y}_{\mathrm{amp}}^{(\ell,p)} - \mathcal{G}_{\mathrm{amp}}^{(\ell,p)}(\mathbf{x}) \right\|_{\mathrm{L2}}^{2} + \alpha \cdot \mathrm{TV}(\mathbf{x}) \tag{S4}$$

Here, $\mathbf{y}_{\mathrm{amp}}^{(\ell,p)}$ and $\mathcal{G}_{\mathrm{amp}}^{(\ell,p)}(\mathbf{x})$ denote the scattering *amplitude* measurement and the MSBP forward model prediction of the scattering field measurement, respectively, for the ℓ-th plane-wave illumination angle and p-th sample-defocus position. Recall from the main manuscript that defocused amplitude measurements were generated by numerically propagating the complex-valued fields, each captured under angular illumination by the interferometric system, to varying defocus distances. L and P denote the total number of illumination angles and sample defocus positions, respectively. $\|\cdot\|_{\mathrm{L2}}$ denotes the L2-norm operator. The term $\mathrm{TV}(\cdot)$ denotes the 3D total-variation regularizer applied via proximal operator. The general minimization procedure is described step-by-step below, and follows the methodology outlined by Chowdhury *et al.* [2] (with minor modifications related to the complex gradient):

(1) Determine the plane-wave illumination fields $\mathbf{E}_0^{(\ell)}$ for each angle ℓ, which can be efficiently computed using standard angle-calibration methods [5].

(2) Initialize an M-layer reconstruction volume with constant starting RI value n_m. This will serve as the initial estimate of $\mathbf{x}$.

(3) Simulate the forward field through the M-layer reconstruction volume under illumination angle ℓ, propagating between layers using the MSBP model. Then propagate the resulting exit field in free space to defocus plane p.

(4) Compute the gradient term and use it for one update step to refine the estimate for the sample's 3D RI distribution (see **Supplementary Sections 1.3, 1.4).**

(5) Repeat steps 3 and 4 for each angle ℓ and defocus plane p.

(6) After the gradient update is applied for each angle ℓ and defocus plane *p,* apply reconstruction priors (see **Supplementary Section 1.5**).

(7) Apply a momentum step to accelerate convergence of the volumetric estimate of the sample's RI [6].

(8) Repeat steps 3-7**,** cycling over all illumination angles and defocus planes, until convergence. Convergence is determined by a maximum iteration count or a stopping criterion based on the per-iteration change in loss falling below a predefined threshold.

1.3 Gradient for complex-valued RI using field-based loss function

We present here a rigorous derivation of the loss gradient, beginning with the complex-valued gradient of the *field*-based loss function. Although the reconstruction algorithm in Eq. (S4) uses an amplitude-only loss during optimization, the field-based formulation provides a simpler intermediate derivation. The extension to amplitude-based loss is described in the next section **Supplementary Section 1.4**.

The field-based loss function quantifies the discrepancy between measured complex-valued *fields* and the *field* predictions from the MSBP model, and is defined as:

$$\mathcal{L}_{\text{field}}^{(\ell,p)}(\mathbf{x}) = \left\| \ \mathbf{y}_{\text{field}}^{(\ell,p)} - \mathcal{G}_{\text{field}}^{(\ell,p)}(\mathbf{x}) \ \right\|_{\text{L2}}^{2} \tag{S5}$$

Here, $\mathbf{y}_{\text{field}}^{(\ell,p)}$ denotes the scattering *field* measurement corresponding to the ℓ-th plane-wave illumination and p-th sample-defocus position. As before, $\mathcal{G}^{(\ell,p)}(\mathbf{x})$ denotes the MSBP forward model prediction of the scattering field measurement given some volumetric estimate of the sample's RI, **x**. Importantly, in this work, **x** is *complex-valued*, where the real component corresponds to the traditional RI while the imaginary component corresponds to absorptivity.

For notational simplicity, we consider now a single illumination angle and sample-defocus position, thus dropping the indices ℓ and p in future mathematical steps. This is possible because the loss function in Eq. (S4) comprises independent least-squares terms for each illumination angle and defocus position. Minimizing the sum is equivalent to minimizing each term individually, so the gradient derivation for a single angle-position pair generalizes to the full problem.

Recall from Eq. (3) in the main manuscript that:

$$\mathcal{G}_{\text{field}}(\mathbf{x}) = \mathbf{H}_{\Delta\hat{z}} \cdot \mathbf{E}_{M}(\mathbf{x}) \tag{S6}$$

where $\mathbf{E}_M$ denotes the MSBP model's prediction of the field exiting the final (i.e., M-th) sample layer. Matrix multiplication by $\mathbf{H}_{\Delta\hat{z}}$ back-propagates this field by a distance $\Delta\hat{z}$ to the in-focus imaging plane within the sample, while simultaneously applying spatial-frequency filtering to enforce a diffraction-limited pupil function.

Using the identity $\|\mathbf{a}\|_{\text{L2}}^2 = \mathbf{a}^H\mathbf{a}$, we can expand Eq. (S5) above:

$$\mathcal{L}_{\text{field}}(\mathbf{x}) = \left(\mathbf{y}_{\text{field}} - \mathcal{G}_{\text{field}}(\mathbf{x})\right)^{H}\left(\mathbf{y}_{\text{field}} - \mathcal{G}_{\text{field}}(\mathbf{x})\right) \tag{S7}$$

Here, the *H* superscript represents the Hermitian conjugate transpose. Expanding this equation and combining with Eq. (S6), we obtain:

$$\mathcal{L}_{\text{field}}(\mathbf{x}) = \mathbf{y}_{\text{field}}^H \mathbf{y}_{\text{field}} - \mathbf{y}_{\text{field}}^H \cdot \mathbf{H}_{\Delta\hat{z}} \cdot \mathbf{E}_M(\mathbf{x}) - \mathbf{E}_M^H(\mathbf{x}) \cdot \mathbf{H}_{\Delta\hat{z}}^H \cdot \mathbf{y}_{\text{field}} + \mathbf{E}_M^H(\mathbf{x}) \cdot \mathbf{E}_M(\mathbf{x}) \quad \text{(S8)}$$

Note that to get the fourth term, $\mathbf{H}_{\Delta\hat{z}}$ gets matrix multiplied by its Hermitian $\mathbf{H}_{\Delta\hat{z}}^H$, which corresponds to a sequence of forward-and-backwards propagation steps resulting in no propagation. For notational clarity, we exclude the effects of the additional diffraction-limited spatial-filtering, which do not change steps in the derivation.

The expanded form in Eq. (S8) separates the measurement term ($\mathbf{y}_{\text{field}}^H \mathbf{y}_{\text{field}}$) from the other model-dependent terms, which is useful when computing derivatives.

1.3.1. Real-Valued Gradient Reference

When the RI is constrained to be real-valued, the gradient of the loss can be computed using *standard real calculus* [2]:

$$\nabla \mathcal{L}_{\text{field}}(\mathbf{x}) = \frac{\partial \mathcal{L}_{\text{field}}(\mathbf{x})}{\partial \mathbf{x}} \quad \text{(S9)}$$

Here, the gradient is defined as derivative of the scalar and real-valued loss-function $\mathcal{L}_{\text{field}}$ with respect to $\mathbf{x}$, which is often considered real-valued. In such cases, we can expand the derivative in Eq. (S9) above by substituting in the expression for $\mathcal{L}_{\text{field}}$ from Eq. (S8). Conveniently, the first term in Eq. (S8) is independent of $\mathbf{x}$ (i.e., $\partial\ (\mathbf{y}_{\text{field}}^H \mathbf{y}_{\text{field}})/\partial \mathbf{x}\ = 0$). Furthermore, the second and third terms are Hermitian conjugates, so their sum simplifies to twice their real component. This allows the differentiation of Eq. (S8) to be simplified, yielding the classical MSBP gradient expression shown earlier by Kamilov *et al.* [3]:

$$\begin{aligned} \nabla \mathcal{L}_{\text{field}}(\mathbf{x}) &= -2 \cdot \frac{\partial}{\partial \mathbf{x}} \operatorname{Re}\{\mathbf{y}_{\text{field}}^H \cdot \mathbf{H}_{\Delta\hat{z}} \cdot \mathbf{E}_M(\mathbf{x})\} + \frac{\partial}{\partial \mathbf{x}} \left(\mathbf{E}_M^H(\mathbf{x}) \mathbf{E}_M(\mathbf{x})\right) \\ &= -2 \cdot \operatorname{Re}\left\{\mathbf{y}_{\text{field}}^H \cdot \mathbf{H}_{\Delta\hat{z}} \cdot \frac{\partial}{\partial \mathbf{x}} \mathbf{E}_M(\mathbf{x})\right\} + 2 \cdot \operatorname{Re}\left\{\mathbf{E}_M^H(\mathbf{x}) \frac{\partial}{\partial \mathbf{x}} \mathbf{E}_M(\mathbf{x})\right\} \\ &= 2 \cdot \operatorname{Re}\left\{(\mathbf{E}_M(\mathbf{x}) - \mathbf{H}_{\Delta\hat{z}}^H \cdot \mathbf{y}_{\text{field}})^H \cdot \frac{\partial}{\partial \mathbf{x}} \mathbf{E}_M(\mathbf{x})\right\} \end{aligned} \quad \text{(S10)}$$

For more convenience, the relation in Eq. (S6) can be used to express this gradient term in (S10) directly in terms of the measured field $\mathbf{y}_{\text{field}}$ and the MSBP model-prediction $\mathcal{G}_{\text{field}}$:

$$\nabla \mathcal{L}_{\text{field}}(\mathbf{x}) = 2 \cdot \operatorname{Re}\left\{(\mathcal{G}_{\text{field}}(\mathbf{x}) - \mathbf{y}_{\text{field}})^H \cdot \mathbf{H}_{\Delta\hat{z}} \cdot \frac{\partial}{\partial \mathbf{x}} \mathbf{E}_M(\mathbf{x})\right\} \quad \text{(S11)}$$

The quantity inside the parentheses (i.e., $\mathcal{G}_{\text{field}}(\mathbf{x}) - \mathbf{y}_{\text{field}}$) represents the data-residual term, which measures the difference between model-predicted and measured field.

1.3.2. Extension to Complex-Valued RI

In the present work, the sample's RI is *complex-valued*, i.e., $\mathbf{x} = \mathbf{n} + j\mathbf{k}$ where $\mathbf{n}$ represents the traditional RI and $\mathbf{k}$ represents absorptivity. In this case, the real-valued gradient derivation outlined in Eq. (S10) cannot be applied directly to the loss function in Eq. (S8) with complex-valued $\mathbf{x}$. The difficulty arises because *derivatives with respect to a complex variable are only defined for holomorphic functions*, which must satisfy the Cauchy-Riemann conditions. In the case when $\mathbf{x}$ is complex-valued, the real-valued loss function in Eq. (S8) depends on *both* $\mathbf{x}$ and its complex conjugate (described in detail later). Such a function does *not* satisfy the Cauchy-Riemann conditions and is thus *not holomorphic* [1]. Consequently, the complex derivative, $d\mathcal{L}_{\text{field}}(\mathbf{x})/d\mathbf{x}$ does not exist in traditional form.

One possible solution is to reformulate the loss function, originally defined in terms of only $\mathbf{x}$ in Eqs. (S5) and (S7), as a joint-variable function over its real and imaginary components, i.e., $\mathcal{L}_{\text{field}}(\mathbf{n}, \mathbf{k})$. In this case, the gradients with respect to both $\mathbf{n}$ and $\mathbf{k}$, which are individually real-valued, can be taken separately:

$$\begin{aligned} \frac{\partial \mathcal{L}_{\text{field}}(\mathbf{n}, \mathbf{k})}{\partial \mathbf{n}} &= \text{Re}\left\{(\mathcal{G}_{\text{field}}(\mathbf{n}, \mathbf{k}) - \mathbf{y}_{\text{field}})^H \cdot \mathbf{H}_{\Delta \hat{z}} \cdot \frac{\partial}{\partial \mathbf{n}} \mathbf{E}_M(\mathbf{n}, \mathbf{k})\right\} \\ \frac{\partial \mathcal{L}_{\text{field}}(\mathbf{n}, \mathbf{k})}{\partial \mathbf{k}} &= \text{Re}\left\{(\mathcal{G}_{\text{field}}(\mathbf{n}, \mathbf{k}) - \mathbf{y}_{\text{field}})^H \cdot \mathbf{H}_{\Delta \hat{z}} \cdot \frac{\partial}{\partial \mathbf{k}} \mathbf{E}_M(\mathbf{n}, \mathbf{k})\right\} \end{aligned} \quad \text{(S12)}$$

However, such a formulation requires maintaining two independent derivatives and obscures the compact structure of the complex-valued forward model.

1.3.3. Wirtinger Gradient Formulation

Using the Wirtinger framework, derivatives of real-valued functions of complex-valued variables can be expressed in terms of the partial derivatives with respect to the variables and their complex conjugates (i.e., Eq. (S1)) [1]. Based on this formulation, the field-based loss function in Eqs. (S5) and (S7) can be expressed as $\mathcal{L}_{\text{field}}(\mathbf{x}, \mathbf{x}^*)$, where $\mathbf{x}$ and its complex conjugate $\mathbf{x}^*$ are treated as *independent* variables (i.e., $\partial \mathbf{x}/\partial \mathbf{x}^* = \partial \mathbf{x}^*/\partial \mathbf{x} = 0$). The field-based loss, now expressed as a function of both $\mathbf{x}$ and $\mathbf{x}^*$, can now be differentiated with respect to $\mathbf{x}$ for the gradient calculation:

$$\nabla \mathcal{L}_{\text{field}}(\mathbf{x}, \mathbf{x}^*) = \frac{\partial \mathcal{L}_{\text{field}}(\mathbf{x}, \mathbf{x}^*)}{\partial \mathbf{x}} \quad \text{(S13)}$$

Before taking this derivative, we first express $\mathcal{L}_{\text{field}}$ as a function of $\mathbf{x}$ and $\mathbf{x}^*$. Inspecting the expanded form of $\mathcal{L}_{\text{field}}$ in Eq. (S8), we see that the only terms with any dependence on $\mathbf{x}$ are $\mathbf{E}_M$ or $\mathbf{E}_M^H$. We now examine this in more detail.

Recall from Eqs. (1) and (2) in the main text that the field exiting the m-th sample layer can be expressed as:

$$\mathbf{E}_m = \text{diag}(\mathbf{T}_m) \cdot \mathbf{H}_{\Delta z} \cdot \mathbf{E}_{m-1} \quad \text{(S14)}$$

where $\mathbf{H}_{\Delta z}$ denotes optical propagation across a distance Δz between sample layers, and $\mathbf{T}_m$ is the m-th sample layer's transmittance function, given by:

$$\mathbf{T}_m = \exp\left(j \frac{2\pi}{\lambda} \Delta z \, (\mathbf{x}_m - n_0)\right) \quad \text{(S15)}$$

Notably, $\mathbf{T}_m$ is dependent on $\mathbf{x}_m = \mathbf{n}_m + j\mathbf{k}_m$, which is the sample's complex-valued RI at the m-th layer. Thus, the transmittance function is a function of *only* $\mathbf{x}$, i.e., $\mathbf{T}_m = \mathbf{T}_m(\mathbf{x})$. It naturally follows that the field exiting *any* sample layer, including the last M-th layer, can be expressed in terms of *only* $\mathbf{x}$, i.e., $\mathbf{E}_M = \mathbf{E}_M(\mathbf{x})$. This property is the same was what was considered in other works when $\mathbf{x}$ was real-valued [3].

Now, consider the same analysis for $\mathbf{E}_M^H$. By taking the Hermitian transpose of both sides of main text's Eq. (1) and identifying that the Hermitian transpose of a diagonal matrix is equal to simply the original matrix's complex conjugate, it naturally follows that:

$$\mathbf{E}_m^H = \mathbf{E}_{m-1}^H \cdot \mathbf{H}_{\Delta z}^H \cdot \text{diag}(\mathbf{T}_m^*) \quad \text{(S16)}$$

Here, $\mathbf{T}_m^*$ denotes the complex conjugate of $\mathbf{T}_m$. To observe the effects of this conjugate operation, we first decompose $\mathbf{T}_m$ into its absorptive and phase components (as shown earlier in Eq. (2) of main text) and then take the complex conjugate:

$$\begin{aligned}\mathbf{T}_m &= e^{j(2\pi/\lambda)\cdot\Delta z\cdot(\mathbf{x}_m - n_0)} \\ &= e^{-(2\pi/\lambda)\cdot\Delta z\cdot\mathbf{k}_m} \cdot e^{j(2\pi/\lambda)\cdot\Delta z\cdot(\mathbf{n}_m - n_0)}\end{aligned} \tag{S17}$$

$$\begin{aligned}\mathbf{T}_m^* &= e^{-(2\pi/\lambda)\cdot\Delta z\cdot\mathbf{k}_m} \cdot e^{-j(2\pi/\lambda)\cdot\Delta z\cdot(\mathbf{n}_m - n_0)} \\ &= e^{-j(2\pi/\lambda)\cdot\Delta z\cdot(\mathbf{x}_m^* - n_0)}\end{aligned} \tag{S18}$$

Importantly, we see from Eq. (S18) that the conjugate of the transmittance function is a function of *only* the conjugate variable $\mathbf{x}^*$, i.e., $\mathbf{T}_m^* = \mathbf{T}_m^*(\mathbf{x}^*)$. From Eq. (S16), it naturally follows that the Hermitian (or even just simple conjugate) of the field exiting any sample layer, including the last M-th layer, can be expressed in terms of *only* $\mathbf{x}^*$, i.e., $\mathbf{E}_M^H = \mathbf{E}_M^H(\mathbf{x}^*)$. *Notably, this property is observed only when* $\mathbf{x}$ *is considered as a complex-variable.*

Recognizing now that $\mathbf{E}_M$ and $\mathbf{E}_M^H$ depend solely on $\mathbf{x}$ and $\mathbf{x}^*$, respectively, the expansion of $\mathcal{L}_{\text{field}}$ in Eq. (S8) can be rewritten explicitly in terms of $\mathbf{x}$ and $\mathbf{x}^*$:

$$\begin{aligned}\mathcal{L}_{\text{field}}(\mathbf{x},\mathbf{x}^*) = \mathbf{y}_{\text{field}}^H\mathbf{y}_{\text{field}} - \mathbf{y}_{\text{field}}^H \cdot \mathbf{H}_{\Delta\hat{z}} \cdot \mathbf{E}_M(\mathbf{x}) - \mathbf{E}_M^H(\mathbf{x}^*) \cdot \mathbf{H}_{\Delta\hat{z}}^H \cdot \mathbf{y}_{\text{field}} \\ +\mathbf{E}_M^H(\mathbf{x}^*)\mathbf{E}_M(\mathbf{x})\end{aligned} \tag{S19}$$

Based on Eq. (S13), the gradient of $\mathcal{L}_{\text{field}}$ can be computed straightforwardly. We differentiate each term of $\mathcal{L}_{\text{field}}$ by $\mathbf{x}$ and treat any term dependent on $\mathbf{x}^*$ as independent on $\mathbf{x}$, thus contributing zero. Noting that the first and third terms in Eq. (S19) do not depend on $\mathbf{x}$, the gradient is determined solely by the second and fourth terms. Combining with the relation in Eq. (S6), this yields the gradient expression:

$$\nabla\mathcal{L}_{\text{field}}(\mathbf{x},\mathbf{x}^*) = (\mathcal{G}_{\text{field}}(\mathbf{x}) - \mathbf{y}_{\text{field}})^H \cdot \mathbf{H}_{\Delta\hat{z}} \cdot \frac{\partial}{\partial\mathbf{x}}\mathbf{E}_M(\mathbf{x}) \tag{S20}$$

Notably, this gradient expression in Eq. (S20) for a complex-valued $\mathbf{x}$ is closely related that for a real-valued $\mathbf{x}$, shown previously in Eq. (S11). The major differences are simply that: (1) the gradient for the real-valued $\mathbf{x}$ enforces the $\text{Re}\{\cdot\}$ operator, while the gradient for complex-valued $\mathbf{x}$ does not; (2) the complex gradient treats $\mathbf{x}$ and $\mathbf{x}^*$ independently to allow simultaneous optimization over the real and imaginary components; and (3) the derivative term $\partial/\partial\mathbf{x}$ is no longer a real-valued derivative and is instead a generalized derivative from Eq. (S2) incorporating both the real and imaginary components of $\mathbf{x}$, i.e., $\partial/\partial\mathbf{x} = (\partial/\partial\mathbf{n} - j\cdot\partial/\partial\mathbf{k})/2$.

The gradient expression in Eq. (S20) contains the (1) data residual term $\mathcal{G}_{\text{field}}(\mathbf{x}) - \mathbf{y}_{\text{field}}$ measuring the discrepancy between model-predicted and measured fields, which can be readily calculated, and (2) the model sensitivity term $\partial\mathbf{E}_M(\mathbf{x})/\partial\mathbf{x}$, which predicts how the field changes with respect to $\mathbf{x}$. This sensitivity term can be efficiently computed by backpropagating the residual term through the sample volume while modulating it with the gradient of each layer's transmittance function (i.e., $\partial\mathbf{T}_m(\mathbf{x})/\partial\mathbf{x}$). For more detailed derivations, we refer the reader to prior works by Kamilov *et al.* [3] and Kim *et al.* [7] Although those works targeted reconstructing real-only $\mathbf{x}$, the major difference in practice to reconstruct complex-valued $\mathbf{x}$ is simply not to enforce the $\text{Re}\{\cdot\}$ operator.

1.4 Gradient for complex-valued RI using amplitude-only loss function

Our previous work by Kim *et al.* [7] demonstrated that employing a complex-valued field loss function can lead to severe phase-wrapping artifacts when reconstructing $\mathbf{x}$ in complex multiple-scattering samples, even in the absence of absorption. In contrast, significantly

improved performance was achieved by minimizing an *amplitude-based* loss function, defined as the discrepancy between the measured scattering *amplitudes* and the *amplitudes* of the model-predicted fields. This formulation offers the added advantage of being usable with non-interferometric systems that do not measure phase, as demonstrated earlier by Chowdhury *et al.* [2] Thus, we use the amplitude-based loss for the reconstruction results presented in this manuscript. Below, we use Wirtinger framework to formulate the gradient of the amplitude-based loss specifically for complex-valued $\mathbf{x}$.

The inverse-solver formulation was introduced earlier in Eq. (S4). From here, the corresponding amplitude-based loss function can be readily identified:

$$\mathcal{L}_{\text{amp}}(\mathbf{x}, \mathbf{x}^*) = \| \ \mathbf{y}_{\text{amp}} - \mathcal{G}_{\text{amp}}(\mathbf{x}, \mathbf{x}^*) \ \|_{\text{L2}}^2 \tag{S21}$$

Here, $\mathbf{y}_{\text{amp}}$ and $\mathcal{G}_{\text{amp}}$ represent the measured scattering *amplitude* (square-root of intensity) and the *amplitude* of the field predicted by the MSBP model, respectively. We express both $\mathcal{L}_{\text{amp}}$ and $\mathcal{G}_{\text{amp}}$ as functions of $\mathbf{x}$ and $\mathbf{x}^*$ to adhere to the Writinger format. Notably, $\mathcal{G}_{\text{amp}}$ depends on $\mathbf{x}^*$ as well as $\mathbf{x}$ because the magnitude of the predicted field involves the product of the field with its complex conjugate, thereby introducing dependence on the conjugate sample term $\mathbf{x}^*$.

As before, our objective is to compute the gradient of this amplitude loss function using a Writinger derivative with respect to $\mathbf{x}$, while treating $\mathbf{x}^*$ as an independent variable:

$$\nabla\mathcal{L}_{\text{amp}}(\mathbf{x}, \mathbf{x}^*) = \frac{\partial \mathcal{L}_{\text{amp}}(\mathbf{x}, \mathbf{x}^*)}{\partial \mathbf{x}} \tag{S22}$$

Similar to our earlier strategy from before to compute the gradient, we first expand Eq. (S21):

$$\begin{aligned}\mathcal{L}_{\text{amp}}(\mathbf{x}, \mathbf{x}^*) = \mathbf{y}_{\text{amp}}^H \mathbf{y}_{\text{amp}} - \mathbf{y}_{\text{amp}}^H \cdot \mathcal{G}_{\text{amp}}(\mathbf{x}, \mathbf{x}^*) - \mathcal{G}_{\text{amp}}^H(\mathbf{x}, \mathbf{x}^*) \cdot \mathbf{y}_{\text{amp}} \\ + \mathcal{G}_{\text{amp}}^H(\mathbf{x}, \mathbf{x}^*) \cdot \mathcal{G}_{\text{amp}}(\mathbf{x}, \mathbf{x}^*)\end{aligned} \tag{S23}$$

Importantly, we note that both $\mathbf{y}_{\text{amp}}$ and $\mathcal{G}_{\text{amp}}$ are fully real-valued. Thus, Hermitian conjugate transpose operations become equivalent to standard matrix transpose operations, which we denote by the T superscript to follow popular convention. Using the additional matrix identity that $\mathbf{a}^T\mathbf{b} = \mathbf{b}^T\mathbf{a}$, Eq. (S23) becomes:

$$\mathcal{L}_{\text{amp}}(\mathbf{x}, \mathbf{x}^*) = \mathbf{y}_{\text{amp}}^T \mathbf{y}_{\text{amp}} - 2\mathbf{y}_{\text{amp}}^T \cdot \mathcal{G}_{\text{amp}}(\mathbf{x}, \mathbf{x}^*) + \mathcal{G}_{\text{amp}}^T(\mathbf{x}, \mathbf{x}^*) \cdot \mathcal{G}_{\text{amp}}(\mathbf{x}, \mathbf{x}^*) \tag{S24}$$

Differentiating both sides with respect to $\mathbf{x}$, we see that the gradient of $\mathcal{L}_{\text{amp}}$ takes the form:

$$\begin{aligned}\nabla\mathcal{L}_{\text{amp}}(\mathbf{x}, \mathbf{x}^*) &= -2\mathbf{y}_{\text{amp}}^T \cdot \frac{\partial}{\partial \mathbf{x}} \mathcal{G}_{\text{amp}}(\mathbf{x}, \mathbf{x}^*) + 2\mathcal{G}_{\text{amp}}^T(\mathbf{x}, \mathbf{x}^*) \cdot \frac{\partial}{\partial \mathbf{x}} \mathcal{G}_{\text{amp}}(\mathbf{x}, \mathbf{x}^*) \\ &= 2 \cdot \left(\mathcal{G}_{\text{amp}}(\mathbf{x}, \mathbf{x}^*) - \mathbf{y}_{\text{amp}}\right)^T \frac{\partial}{\partial \mathbf{x}} \mathcal{G}_{\text{amp}}(\mathbf{x}, \mathbf{x}^*)\end{aligned} \tag{S25}$$

It is insightful to identify the difference between this gradient expression and the one for the field-based cost function in Eq. (S20). To do this, we first rewrite the expression of the derivative term $\partial \mathcal{G}_{\text{amp}}(\mathbf{x}, \mathbf{x}^*)/\partial \mathbf{x}$ in Eq. (S25).

From earlier in this section when $\mathcal{G}_{\text{amp}}$ was introduced as the amplitude of the model-predicted scattered field, we know that $\mathcal{G}_{\text{amp}}(\mathbf{x}, \mathbf{x}^*) = |\mathcal{G}_{\text{field}}(\mathbf{x})|$. From Eq. (S6), it follows that $\mathcal{G}_{\text{amp}}(\mathbf{x}, \mathbf{x}^*) = |\mathbf{H}_{\Delta\hat{z}} \cdot \mathbf{E}_M(\mathbf{x})|$. Due to the magnitude operation, $\mathcal{G}_{\text{amp}}(\mathbf{x}, \mathbf{x}^*)$ is a function of

both the conjugate terms $\mathbf{E}_M$ and $\mathbf{E}_M^*$. Thus, the derivative term $\partial \mathcal{G}_{\text{amp}}(\mathbf{x}, \mathbf{x}^*)/\partial \mathbf{x}$ in Eq. (S25) can be rewritten via the Wirtinger chain rule, as described earlier in Eq. (S3):

$$\frac{\partial}{\partial \mathbf{x}} \mathcal{G}_{\text{amp}}(\mathbf{x}, \mathbf{x}^*) = \frac{\partial \mathcal{G}_{\text{amp}}}{\partial \mathbf{E}_M} \cdot \frac{\partial \mathbf{E}_M}{\partial \mathbf{x}} + \frac{\partial \mathcal{G}_{\text{amp}}}{\partial \mathbf{E}_M^*} \cdot \frac{\partial \mathbf{E}_M^*}{\partial \mathbf{x}} \tag{S26}$$

In the Wirtinger framework, we recall that $\mathbf{E}_M^*$ is only dependent on $\mathbf{x}^*$, not $\mathbf{x}$. Thus, $\partial \mathbf{E}_M^*/\partial \mathbf{x} = 0$ and the second term in Eq. (S26) vanishes. The first term, $\partial \mathcal{G}_{\text{amp}}/\partial \mathbf{E}_M$, can also be expanded:

$$\begin{aligned} \frac{\partial \mathcal{G}_{\text{amp}}(\mathbf{x}, \mathbf{x}^*)}{\partial \mathbf{E}_M(\mathbf{x})} &= \frac{\partial |\mathbf{H}_{\Delta \hat{z}} \cdot \mathbf{E}_M(\mathbf{x})|}{\partial \mathbf{E}_M(\mathbf{x})} \\ &= \frac{1}{2} \operatorname{diag}\left(\frac{\mathbf{H}_{\Delta \hat{z}} \cdot \mathbf{E}_M(\mathbf{x})}{|\mathbf{H}_{\Delta \hat{z}} \cdot \mathbf{E}_M(\mathbf{x})|} \right)^H \cdot \mathbf{H}_{\Delta \hat{z}} \end{aligned} \tag{S27}$$

We recall again from Eq. (S6) that $\mathcal{G}_{\text{field}}(\mathbf{x}) = \mathbf{H}_{\Delta \hat{z}} \cdot \mathbf{E}_M(\mathbf{x})$. Thus, Eq. (S27) can be expressed in terms of the model prediction of the scattered *fields*:

$$\begin{aligned} \frac{\partial \mathcal{G}_{\text{amp}}(\mathbf{x}, \mathbf{x}^*)}{\partial \mathbf{E}_M(\mathbf{x})} &= \frac{1}{2} \operatorname{diag}\left(\frac{\mathcal{G}_{\text{field}}(\mathbf{x})}{|\mathcal{G}_{\text{field}}(\mathbf{x})|} \right)^H \cdot \mathbf{H}_{\Delta \hat{z}} \\ &= \frac{1}{2} \operatorname{diag}\left(e^{-j \angle \mathcal{G}_{\text{field}}(\mathbf{x})} \right) \cdot \mathbf{H}_{\Delta \hat{z}} \end{aligned} \tag{S28}$$

Here, $\angle \mathbf{z}$ denotes the phase component of some complex-valued vector $\mathbf{z}$. Substituting Eqs. (S26) and (S28) into the $\partial \mathcal{G}_{\text{amp}}(\mathbf{x}, \mathbf{x}^*)/\partial \mathbf{x}$ in Eq. (S25), we can re-express the gradient for $\mathcal{L}_{\text{amp}}$:

$$\begin{aligned} \nabla \mathcal{L}_{\text{amp}}(\mathbf{x}, \mathbf{x}^*) &= 2 \cdot \left(\mathcal{G}_{\text{amp}}(\mathbf{x}, \mathbf{x}^*) - \mathbf{y}_{\text{amp}} \right)^T \frac{\partial}{\partial \mathbf{x}} \mathcal{G}_{\text{amp}}(\mathbf{x}, \mathbf{x}^*) \\ &= \left(\mathcal{G}_{\text{amp}}(\mathbf{x}, \mathbf{x}^*) - \mathbf{y}_{\text{amp}} \right)^T \operatorname{diag}\left(e^{-j \angle \mathcal{G}_{\text{field}}(\mathbf{x})} \right) \cdot \mathbf{H}_{\Delta \hat{z}} \cdot \frac{\partial \mathbf{E}_M}{\partial \mathbf{x}} \end{aligned} \tag{S29}$$

Further simplification is obtained by incorporating the complex-valued term $\operatorname{diag}\left(e^{-j \angle \mathcal{G}_{\text{field}}(\mathbf{x})} \right)$ into the transposed grouping of $\mathcal{G}_{\text{amp}} - \mathbf{y}_{\text{amp}}$. This operation conjugates the exponential term and converts the transpose into a Hermitian transpose. Moreover, because $\mathcal{G}_{\text{amp}} = |\mathcal{G}_{\text{field}}|$, multiplying $\mathcal{G}_{\text{amp}}$ by the complex exponential of the phase of $\mathcal{G}_{\text{field}}$ returns the field itself, i.e., $\mathcal{G}_{\text{field}} = \mathcal{G}_{\text{amp}} e^{-j \angle \mathcal{G}_{\text{field}}}$. With these simplifications, Eq. (S29) can be rewritten as:

$$\nabla \mathcal{L}_{\text{amp}}(\mathbf{x}, \mathbf{x}^*) = \left(\mathcal{G}_{\text{field}}(\mathbf{x}) - \operatorname{diag}\left(e^{-j \angle \mathcal{G}_{\text{field}}(\mathbf{x})} \right) \cdot \mathbf{y}_{\text{amp}} \right)^H \cdot \mathbf{H}_{\Delta \hat{z}} \cdot \frac{\partial \mathbf{E}_M}{\partial \mathbf{x}} \tag{S30}$$

The gradient of the *amplitude*-based loss $\mathcal{L}_{\text{amp}}$ can thus be expressed in terms of $\mathcal{G}_{\text{field}}$, which is the forward model for complex-valued *fields*. Notably, this relationship closely mirrors the gradient of $\mathcal{L}_{\text{field}}$ in Eq. (S20). The *only* difference lies in the residual term in Eq. (S30), where the *amplitude* measurement $\mathbf{y}_{\text{amp}}$ is combined with the *phase* component of $\mathcal{G}_{\text{field}}$, effectively transforming it into a synthetic complex-valued field measurement. *Computing* $\partial \mathbf{E}_{\text{M}}/\partial \mathbf{x}$ *follows exactly the same recursive backpropagation procedure as in the field-based MSBP model* (see derivation of this procedure in Kim *et al.* [7]).

1.5 Constraining the reconstruction

The loss landscape of the MSBP problem is non-convex, making the optimization susceptible to convergence at local minima. To promote convergence and ensure physically meaningful reconstructions, we incorporate specific constraints and regularization into the inverse-solver.

First, we enforced consistency bounds on the reconstructed distribution for $\mathbf{x}$. Specifically, since the sample is assumed to exhibit no optical gain, the absorptivity was constrained to be non-negative, i.e., $\mathbf{k} = \mathrm{Im}\{\mathbf{x}\} \geq 0$.

Second, we imposed a structural smoothness prior via total variation (TV) regularization, applied in a proximal framework [8]. In other inverse-scattering works where $\mathbf{x}$ was assumed to be fully real-valued, proximal TV regularization was shown to mitigate the missing-cone problem, model mismatch, and measurement noise [3,9]. With complex-valued RI, proximal TV was applied independently to $\mathbf{n}$ and $\mathbf{k}$. Their respective regularization strengths were tuned separately to optimize quantitative reconstruction accuracy.

Once all stochastic gradient steps to update $\mathbf{x}$ had been completed across the full set angular and defocus measurements, the proximal TV operator was applied as a subsequent update step to the current estimate of $\mathbf{x}$:

$$\begin{aligned} \mathbf{n} &\leftarrow \mathrm{prox}_{\alpha}(\mathbf{n}) \\ \mathbf{k} &\leftarrow \mathrm{prox}_{\beta}(\mathbf{k}) \end{aligned} \tag{S31}$$

Here, $\mathrm{prox}_{\gamma}(\cdot)$ denotes the proximal operator for TV regularization, defined as:

$$\mathrm{prox}_{\gamma}\{\mathbf{a}\} = \underset{\hat{\mathbf{x}}}{\mathrm{argmin}} \left\{ \frac{1}{2} \|\mathbf{a} - \hat{\mathbf{a}}\|_{L2}^{2} + \gamma \cdot \mathrm{TV}(\hat{\mathbf{a}}) \right\} \tag{S32}$$

where $\mathrm{TV}(\cdot)$ denotes the 3D TV norm and γ represents the strength of regularization.

S2. Computational details

Here, we describe the software environment used for computational reconstructions and summarize the corresponding reconstruction times.

Reconstructions were performed using a custom MATLAB implementation of the MSBP inverse-scattering solver. Computations were run in MATLAB R2024b with GPU acceleration. Most reconstructions were performed on a workstation equipped with an NVIDIA RTX 6000 Ada Generation GPU and an Intel Xeon w5-2545 CPU operating at 3.50 GHz. For the zebrafish embryo dataset, which consisted of 24 fields of view, reconstructions were performed on the Lonestar6 supercomputer at the Texas Advanced Computing Center (TACC), enabling parallel processing of several individual fields of view.

There are two major implementation differences when implementing MSBP with complex-valued gradients versus the real-only gradients. These are intrinsic to the complex nature of the reconstruction because the optimization can now be thought of as adding another dimension in the imaginary domain. One factor that increases the compute time is during regularization of the complex volume. Here, the real and imaginary components are fed separately through a proximal TV function (e.g., Eq. (S31)), which approximately doubles the computation time of the regularization step when compared to the standard model. Second, standard MSBP uses single- or double-precision floats (32 or 64 bits). To store complex-valued distributions, the memory requirements of the reconstructed object and accompanying gradients double as the real and imaginary components both require a respective 32 or 64 bits. We compare the real- and complex-valued implementations of MSBP in **Table S2.1** below:

**Parameters**		**Sample Type**			
		Microspheres (**Figure 4**)	*Micrasterias Radiata* (**Figure 5**)	Succulent section (**Figure 6**)	48hpf zebrafish embryo (**Figure 7**)
**per field-of-view**	# of Illumination angles	200	500	400	400
	Numerical aperture (NA)	1.4	0.8	0.8	0.8
	Maximum illumination angle [°]	62.95	53.13	53.13	53.13
	Reconstruction volume [μm × μm × μm]	16.7 × 16.7 × 22.3	138.8 × 138.8 × 79.32	198.2 × 198.2 × 198.2	206 × 206 × 247.2
	Reconstruction size Real/Complex [MB]*	Real: 4.5 Complex: 9	Real: 196 Complex: 392	Real: 1049 Complex: 2097	Real: 1152 Complex: 2304
	Defocus Planes	5	3	3	3
	Number of iterations	50	75	100	100
	Reconstruction time with real gradients (1 iteration) [**s**]	2.97**	14.59**	28.66**	34.40**
	Reconstruction time with complex-valued gradients (1 iteration) [**s**]	2.97**	14.90**	34.21**	46.50**
**Number of fields of view**		1	1	1	24

Table. S2.1. Reconstruction parameters are reported for the 3D RI results presented in Figs. 4 – 7 of the main manuscript. Parameters include the number of illumination angles used for reconstruction, numerical aperture of the imaging lens, maximum illumination angle, and the reconstruction volume dimensions. Furthermore, we include information on how many iterations were used for convergence and how many defocus planes were used to achieve defocus diversity. Lastly, computational reconstruction times are reported for both the real-valued and complex-valued gradient formulations. (*Single precision reconstruction, **Computation times are reported based on a local computer equipped with an NVIDIA RTX 6000 Ada Generation GPU and MATLAB R2024b)

S3. Imaging parameters

To accommodate the differing optical and geometric properties of the samples, imaging parameters were adjusted for each experiment. For the dyed microsphere experiments, a high numerical aperture objective (NA = 1.4) was used to maximize spatial frequency support and enable accurate quantitative estimation of the complex refractive index. For biological samples, the objective numerical aperture was reduced (NA = 0.8) to increase the working distance, improving the ease of imaging for thick biological specimens.

In addition to the objective selection, the number of angular measurements and defocus planes was adjusted depending on sample complexity and experimental constraints. **Table S3.1** summarizes the acquisition parameters used for each dataset.

Sample	Wavelength (nm)	NA	# of angular measurements	# of sample-defocus planes (and sample-defocus positions)
Absorptive microspheres	450, 532, 635	1.4	200	5 ($\pm$80 μm, $\pm$40 μm, and 0 μm)
Micrasterias Radiata (algae)	635	0.8	500	3 ($\pm$10 μm and 0 μm)
Succulent section	635	0.8	400	3 ($\pm$10 μm and 0 μm)
48 hpf zebrafish embryo	635	0.8	400	3 ($\pm$10 μm and 0 μm)

Table S3.1. Imaging parameters are presented here that were used to reconstruct the different samples presented in this work. This includes the number of angular measurements (ℓ), number (p) and position of defocus planes, imaging system's numerical aperture (NA), and illumination wavelengths used for each dataset.

S4. Comparing RI reconstruction using amplitude versus field loss functions

In the main manuscript, 3D RI reconstruction is formulated by minimizing the difference between the measured scattering amplitudes and those predicted by the MSBP forward model. We refer to this as minimizing the *amplitude* loss function:

$$\hat{\mathbf{x}} = \arg\min_{\mathbf{x}} \frac{1}{LP} \sum_{p=1}^{P} \sum_{\ell=1}^{L} \left\| \mathbf{y}_{\text{amp}}^{(\ell,p)} - \left| \mathcal{G}^{(\ell,p)}(\mathbf{x}) \right| \right\|_{\text{L2}}^{2} + \alpha \cdot \text{TV}(\mathbf{x}) \quad \text{(S33)}$$

Here, $\mathbf{y}_{\text{amp}}^{(\ell,p)}$ denotes the measured scattering *amplitude* for the ℓ-th illumination angle and the p-th sample-defocus position, while $\mathcal{G}^{(\ell,p)}(\mathbf{x})$ denotes the MSBP forward-model prediction of the complex-valued field, given an estimate of the sample $\mathbf{x}$. Although the forward model predicts the full complex field, only its amplitude is used within the loss function in Eq. (S36). The remaining notation follows that of the main text.

Because our off-axis interferometric system measures the full complex optical field, one could alternatively formulate the inverse problem by minimizing the discrepancy between the measured and predicted complex-valued scattered *fields*. We refer to this as minimizing the *field* loss function:

$$\hat{\mathbf{x}} = \arg\min_{\mathbf{x}} \frac{1}{LP} \sum_{p=1}^{P} \sum_{\ell=1}^{L} \left\| \mathbf{y}_{\text{amp}}^{(\ell,p)} - \left| \mathcal{G}^{(\ell,p)}(\mathbf{x}) \right| \right\|_{\text{L2}}^{2} + \alpha \cdot \text{TV}(\mathbf{x}) \quad \text{(S34)}$$

Here, $\mathbf{y}_{\text{field}}^{(\ell)}$ denotes the measured scattering field corresponding to the ℓ-th plane-wave illumination. Unlike Eq. (S36), this formulation directly penalizes errors in *both* amplitude and phase. Furthermore, because a measured complex field can be numerically propagated to arbitrary defocus planes, additional defocus measurements are unnecessary when using the field-based formulation.

At first glance, minimizing the field loss function in Eq. (S34) may appear preferable, since complex-valued field measurements contain more information than amplitude alone. Surprisingly, however, we consistently observe the opposite. This behavior was also discussed in our prior work Kim *et al.* [7], where we hypothesized that phase wrapping introduces ambiguities that can mislead the non-convex inverse solver, causing convergence to erroneous

solutions. To illustrate this phenomenon, we reconstructed the tail of a 24 hpf zebrafish embryo, a multiple-scattering but non-absorptive sample. Because the sample is transparent, reconstruction can be performed using either real-valued or complex-valued RI gradient updates. For each case, we compare the results from minimizing the amplitude (Eq. S36) versus field (Eq. S37) loss functions.

As shown in **Figure S4.1**, minimizing the amplitude loss consistently produced accurate, artifact-free reconstructions regardless of whether real- or complex-valued gradient updates were used. In contrast, minimizing the field loss produced severe reconstruction artifacts (see **Figures S4.1(b,d)**). These artifacts are observed to localize to regions where the measured phase wraps through 2π. Because the field loss directly penalizes complex-valued phase errors, discontinuities introduced by phase wrapping propagate into the reconstructed volume as spurious "wrapped" RI structures. In contrast, the amplitude-based loss is insensitive to phase wrapping, since the measured amplitudes contain no 2π ambiguity. Furthermore, when using complex-valued gradients, we observed that phase-wrapping errors can be misattributed by the inverse solver and leak into the reconstructed absorptivity distribution. This failure mode is avoided when using the amplitude-based loss.

Finally, note that the color scales differ between the reconstructions. The larger dynamic range required for the field-based results reflects the magnitude of the reconstruction artifacts rather than genuine sample contrast.

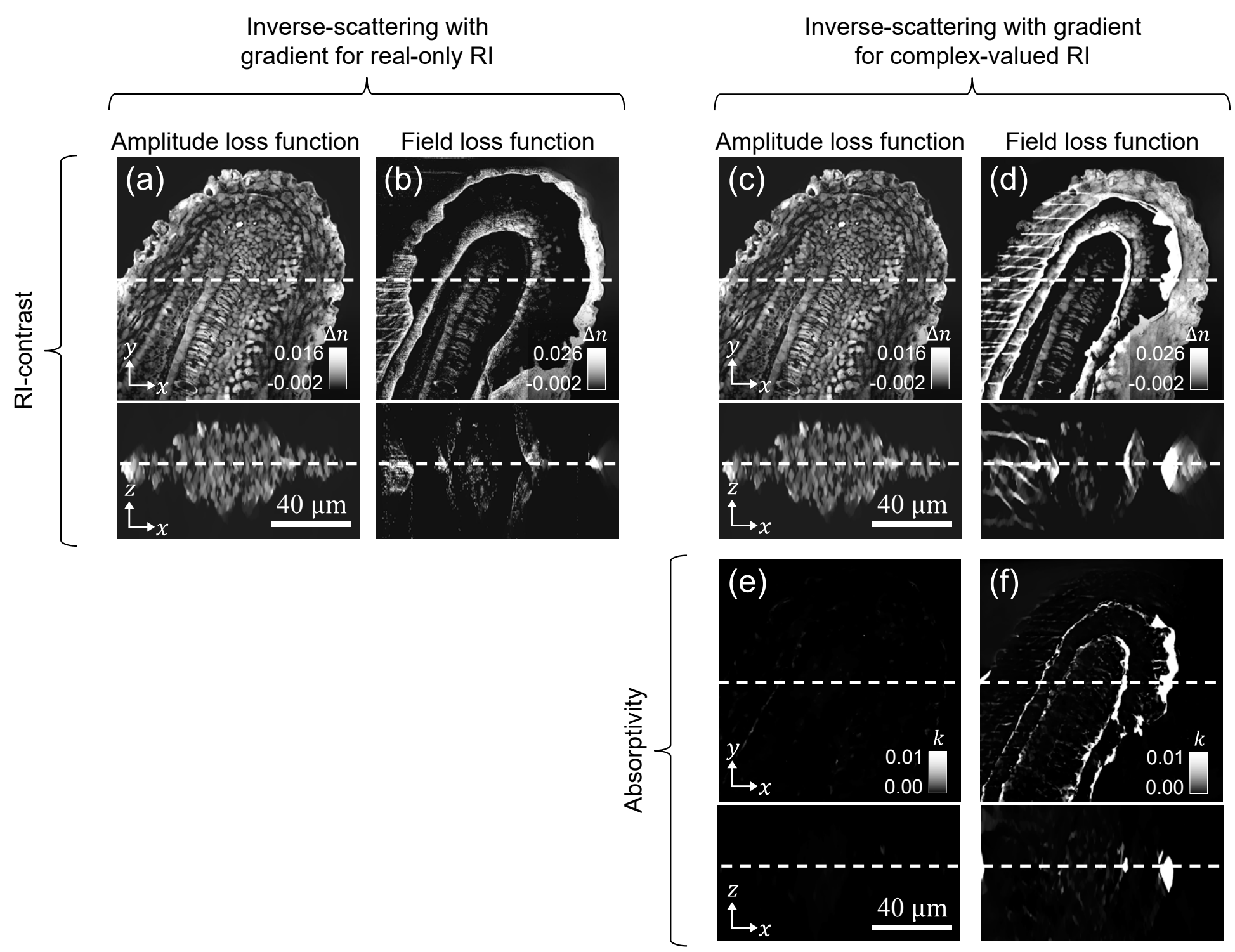

Figure S4.1. Comparison of 3D RI reconstructions of a 24 hpf zebrafish embryo using different gradient updates and loss functions. Reconstructions were performed using **(a,b)** real-valued and **(c–f)** complex-valued RI gradient updates with either (a,c,e) an amplitude-based loss function or (b,d,f) a field-based loss function. Orthogonal xy and xz cross-sections are shown along the dashed white lines.

S5. Quantitative accuracy of reconstructed RI in absorptive microspheres

To quantitatively evaluate our framework, we reconstructed the *complex-valued* RI of *absorptive, multi-color* polystyrene microspheres at multiple (red, green, blue) illumination wavelengths, to test whether the microspheres' (conventional) RI and absorptivity could be

accurately recovered. Reconstruction accuracy was evaluated by comparing the reconstructed RI-contrast (Δn) and absorptivity (k) values to their corresponding "ground-truth" values ($\Delta\hat{n}$ and $\hat{k}$, respectively) extracted directly from the field-based measurements, as described in **Section 4.1** of the main text. Results are shown in **Table S5.1** below.

Notably, signal-to-noise ratio (SNR) was found to be inversely correlated with sample absorptivity. To account for this variation, regularization strengths were tuned independently for each combination of microsphere color and illumination wavelength. While this tuning introduced modest variation in reconstruction accuracy, the recovered RI and absorptivity values showed overall strong agreement with ground-truth across all three illumination wavelengths ($\lambda_b = 450$ nm, $\lambda_g = 532$ nm, $\lambda_r = 635$ nm) and all three microsphere colors (blue, yellow, violet). For example, the absolute reconstruction error for RI and absorptivity was $|\ \Delta n - \Delta\hat{n}\ | < 0.001$ and $|\ k - \hat{k}\ | < 0.002$, respectively, across all tested conditions. This level of accuracy is competitive with the accuracies reported by other RI imaging methods [10-12].

Color	Diameter	λ (nm)	Δn	$\Delta\hat{n}$	$\lvert\Delta n - \Delta\hat{n}\rvert$	k	$\hat{k}$	$\lvert k - \hat{k}\rvert$
Blue	8.54	450	0.0154	0.0149	0.0006	0.0009	0.0010	0.0001
	8.54	532	0.0064	0.0065	0.0001	0.0052	0.0058	0.0006
	8.54	635	0.0207	0.0198	0.0009	0.0280	0.0299	0.0019
Yellow	9.88	450	0.0243	0.0242	0.0001	0.0088	0.0098	0.0010
	9.88	532	0.0210	0.0210	0.0000	0.0001	0.0002	0.0001
	9.88	635	0.0180	0.0180	0.0000	0.0002	0.0002	0.0000
Violet	9.59	450	0.0165	0.0172	0.0007	0.0000	0.0001	0.0001
	9.59	532	0.0163	0.0167	0.0004	0.0006	0.0007	0.0001
	9.59	635	0.0161	0.0162	0.0001	0.0006	0.0006	0.0000

Table S5.1. Quantitative comparison between ground-truth and reconstructed RI + absorptivity for blue, yellow, and violet microspheres illuminated at wavelengths of 450 nm, 532 nm, and 635 nm. The table lists the reconstructed RI-contrast (Δn) and absorptivity (k) as well as their corresponding ground-truth values ($\Delta\hat{n}$ and $\hat{k}$, respectively) alongside microsphere diameters.

S6. Comparison of microsphere inverse-scattering with real-valued vs complex-valued gradient update

In **Figure 4** of the main text, we showed how RI reconstruction of an absorptive microsphere was affected by 1) using the gradient update term derived specifically for complex-valued RI; and 2) using sufficient measurement diversity, combining both angular scattering measurements and various sample-defocus positions. In all cases, the *amplitude* components of the complex-valued fields were used in the inverse-solver (Eq. 4 in the main text). We demonstrated that when using the gradient update for complex-valued RI, both the real (traditional RI) and imaginary (absorptivity) were reconstructed with high quantitative accuracy only when *both* angular illumination and sample-defocus measurements were used in the inverse-solver. When using *only* angular measurements, the real component reconstructed poorly. Thus, angular measurements alone were observed to be insufficient when reconstructing the absorptive sample.

We also showed in **Figure 4** of the main text that the real-valued RI gradient update yielded strong artifacts in the reconstruction that failed to recover the spherical structure of the microsphere. However, that demonstration was limited to the case of using angular illumination measurements alone, leaving open the question of whether incorporating sample-defocus

measurements might naturally remedy these failures. **Figure S6.1** below addresses this directly, showing that reconstructions remained poor with the real-valued RI gradient update even when *both* angular and defocus measurements were included (see **Figure S6.1(e)**). This confirmed that accurate inverse-scattering for 3D absorptive samples requires not only sufficient measurement diversity but also a gradient update explicitly derived to account for absorption.

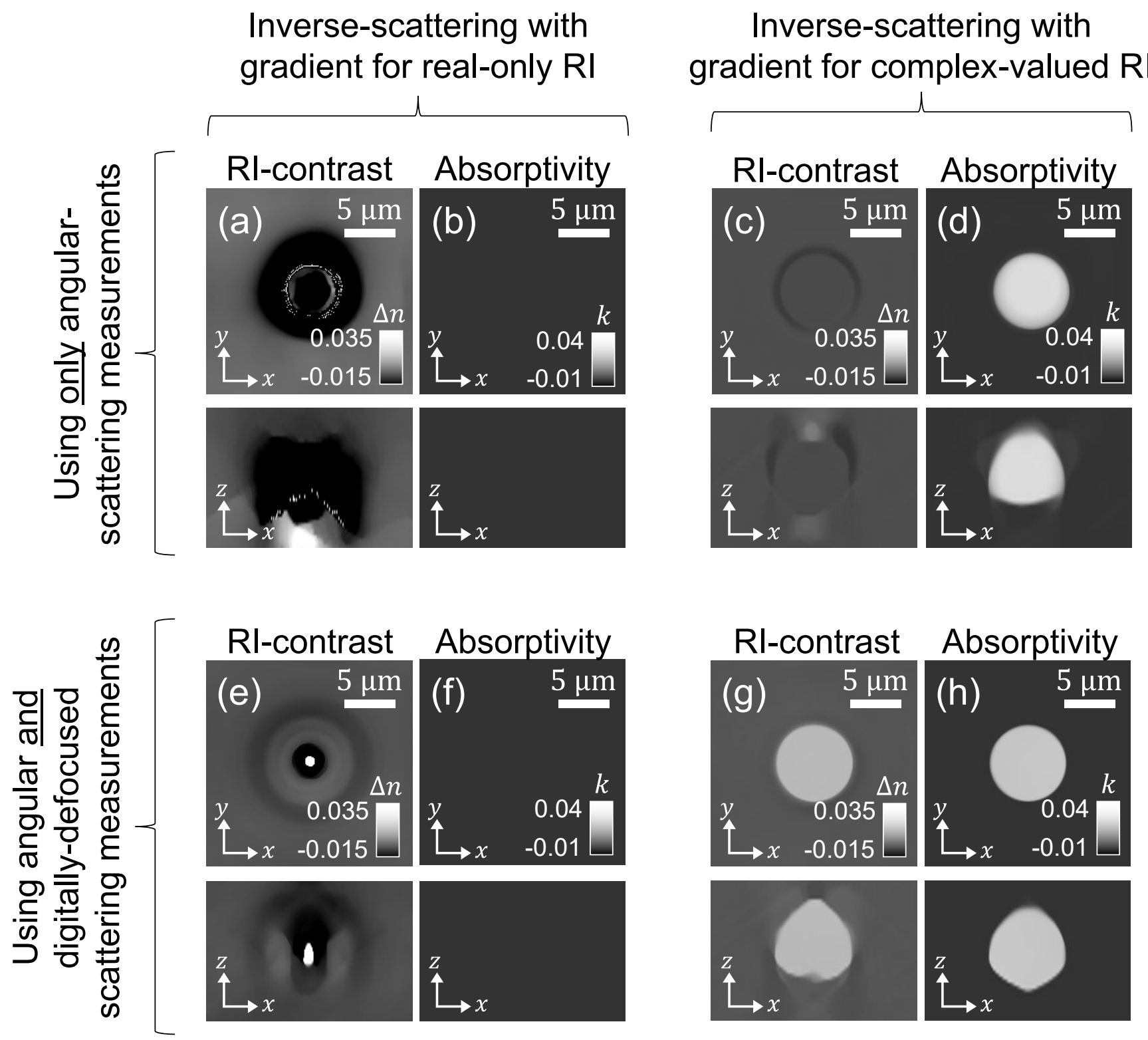

Figure S6.1. Comparison of 3D RI reconstructions of an absorptive microsphere across different measurement and gradient configurations. Results are shown for **(a,b,e,f)** real-valued and **(c,d,g,h)** complex-valued RI gradient updates, and for **(a–d)** angular illumination alone versus **(e–h)** combined angular and sample-defocus measurements. In all cases, an amplitude-only loss function was used within the MSBP inverse-scattering solver. The highest-quality reconstruction was obtained with the complex-valued RI gradient update combined with angular and sample-defocus measurements.

S7. Complex-valued gradient update for reconstructing RI in non-absorptive microspheres

While the preceding sections demonstrated the accuracy of the proposed MSBP formulation for reconstructing the complex-valued RI in *absorptive* samples, we next evaluate its performance on a *non-absorptive* sample. This experiment serves as a negative control to verify that extending the reconstruction to complex-valued RI does not introduce artifacts in the transparent samples considered in our previous works [2,7]. To this end, we reconstructed a non-absorptive polystyrene microsphere using both the original real-valued and the proposed complex-valued gradient formulations. In the absence of absorption, the complex-valued formulation is expected to recover the same real-valued RI distribution as the original model while producing a virtually zero-valued absorptivity map.

Consistent with these expectations, both formulations produced nearly identical RI distributions (see **Figure S7.1** below). When only angular measurements were used, the

complex-valued formulation reconstructed some slight residual non-zero absorptivity values (see yellow arrow in **Figure S7.1(d)**); however, these artifacts disappeared after incorporating digitally refocused measurements. Interestingly, we observed that digital refocusing was not required for achieving accurate RI reconstruction in this non-absorptive microsphere. This contrasts with the absorptive microsphere experiments, where digital refocusing was essential for accurately recovering both the RI and absorptivity distributions.

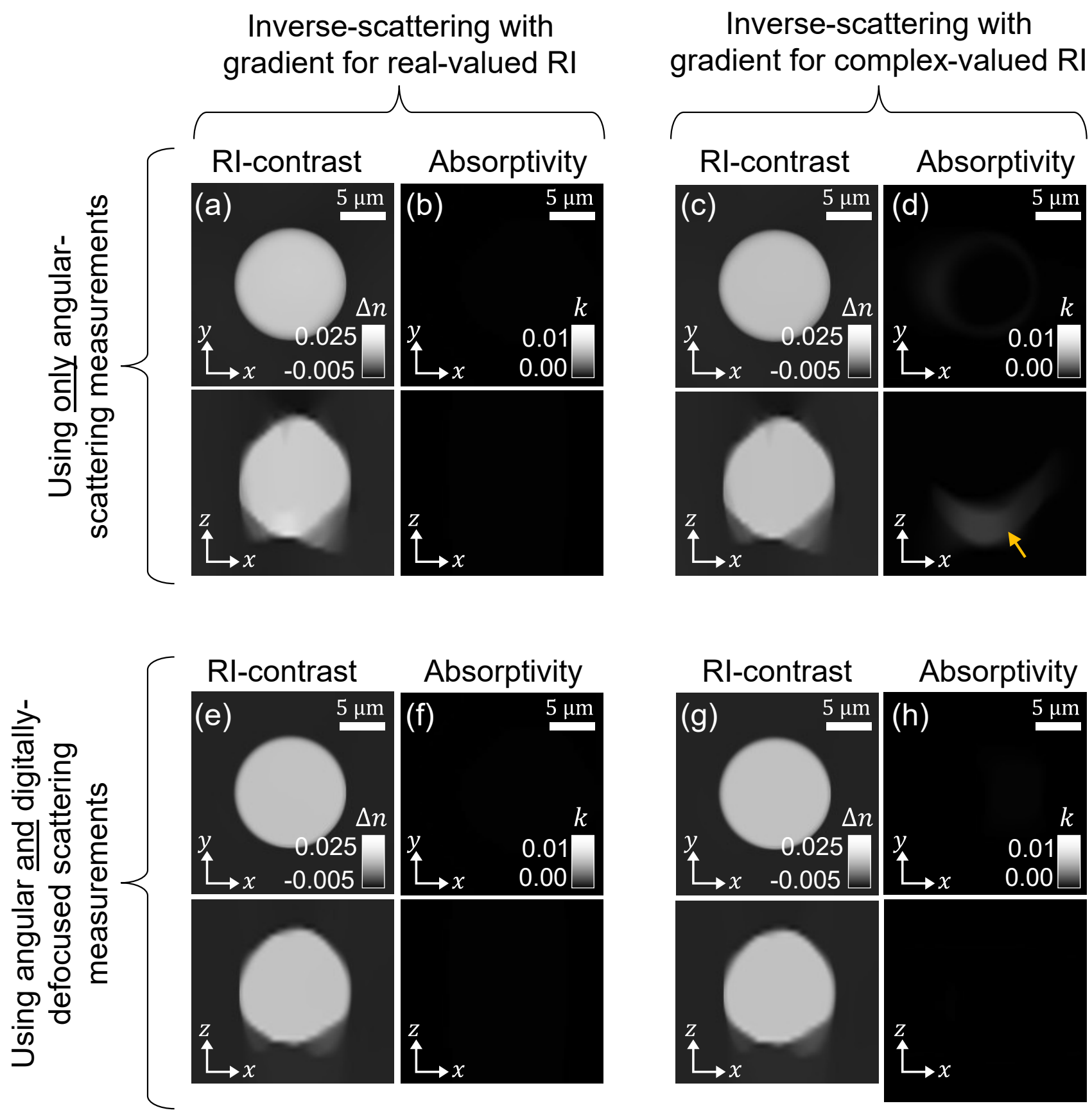

Figure S7.1. Comparison of 3D RI reconstructions of a non-absorptive microsphere under different measurement and reconstruction configurations. Results are shown for **(a,b,e,f)** real-valued and **(c,d,g,h)** complex-valued RI gradient updates, using measurements obtained with **(a – d)** angle-varying illumination alone and **(e – h)** combined angle-varying illumination and sample-defocus. All reconstructions employed an amplitude-only loss function within the MSBP inverse-scattering framework. The real- and complex-valued formulations produced nearly identical RI distributions, while the absorptivity recovered by the complex formulation was nearly zero, as expected for a non-absorptive sample. Small residual absorptivity values were observed when reconstructing from angle-varying illumination alone but disappeared when defocus measurements were included. Interestingly, accurate RI reconstruction of this non-absorptive microsphere was achieved even without defocus, in contrast to the absorptive microspheres, where defocus was essential for accurate recovery of both RI and absorptivity.

S8. Characterizing mean free path in scattering samples

To quantify the degree of scattering in our samples, we estimate the extinction mean free path (MFP) l_{ext} following the approach described by Yasuhiko and Takeuchi [13]. Briefly, this method exploits the angle-scanned complex field measurements that we already acquired for

RI reconstruction – no additional hardware or measurements are required. We first describe the high-level mathematical formulation of this method.

8.1 Mathematical framework

The key idea is to computationally synthesize a focused-point illumination pattern by coherently superposing the angle-scanned fields:

$$\mathbf{p} = \sum_{\ell=1}^{L} \mathbf{y}_{\text{field}}^{(\ell)} \tag{S35}$$

Here, $\mathbf{y}_{\text{field}}^{(\ell)}$ denotes the complex-valued scattering *field* measurement corresponding to the ℓ-th plane-wave illumination.

Eq. (S35) is computed both without the sample ($\mathbf{p}_{\text{B}}$, containing a sharp peak containing only ballistic photons) and with the sample ($\mathbf{p}_{\text{sample}}$, where the peaks are scrambled and attenuated by scattering). The ballistic power transmittance T_{B} is then extracted by projecting $\mathbf{p}_{\text{B}}$ onto $\mathbf{p}_{\text{sample}}$ via an inner product, exploiting the orthogonality of the ballistic and multiply-scattered components for scattering samples:

$$T_{\text{B}} = \frac{\left|\langle \mathbf{p}_{\text{sample}}, \mathbf{p}_{\text{B}} \rangle_A\right|^2}{|\langle \mathbf{p}_{\text{B}}, \mathbf{p}_{\text{B}} \rangle_A|^2} \tag{S36}$$

Here, $\langle \mathbf{m}, \mathbf{n} \rangle = \mathbf{n}^H \mathbf{m}$ denotes the inner product of $\mathbf{m}$ and $\mathbf{n}$, and the Hermitian transpose ^{H} is due to the complex nature of the field variables. The subscript A variable of the inner product represents that the inner product is taken specifically across a region-of-interest A within the measurement.

Finally, the MFP is obtained by inverting the Beer-Lamber law:

$$l_{\text{ext}} = -\frac{L}{\log\, T_{\text{B}}} \tag{S37}$$

where l_{ext} is the extinction MFP computed by measuring the sample thickness L from the reconstructed RI tomogram. We refer the reader to Yasuhiko and Takeuchi [13] for a full derivation and demonstration of this approach.

Note that this *extinction* MFP l_{ext} represents the average distance a photon travels before undergoing *either* a scattering or absorption event. It therefore represents a lower bound on the *scattering* MFP. In the limit of negligible absorption, the extinction and scattering MFPs become identical.

8.2 Characterizing sample scattering

We first used this framework to characterize l_{ext} of a series of scattering phantoms. The phantoms consisted of a cell phantom embedded within scattering cubes of lateral dimensions 40, 60, 80, and 100 µm, each constructed from densely packed horizontal and vertical rods (see **Figures S8.1(a-d)**). The rod network forms a pseudo-homogeneous scattering medium when viewed over regions substantially larger than the smallest structural feature size (300 nm), provided regions containing the larger cell phantom are avoided. Accordingly, we selected analysis regions in the surrounding rod network that we considered approximately homogeneous at the spatial scale used for estimating l_{ext}. Furthermore, because absorption is negligible in these phantoms, the extinction MFP is equal to the scattering MFP.

Figure S8.1 shows representative raw measurements of the phantoms under normal illumination. The yellow rectangles at the top left of the phantoms indicate the regions used for analysis. Each selected region was further divided into adjacent subregions, each serving as a

region A for estimating l_{ext} using Eqs. (S3)-(S4). For a homogeneous scattering medium, l_{ext} is an intrinsic material property and should therefore remain approximately constant as sample thickness increases, provided the additional thickness consists of the same underlying random medium.

This behavior is indeed observed. As shown in **Figure S8.1(e)**, all four phantoms yielded consistent distributions for l_{ext}, with mean and standard-deviation values of approximately 25 µm and 7.5 µm, respectively, despite the phantoms' differing dimensions. The agreement across all phantom sizes indicates that the proposed framework provides stable estimates of l_{ext}. Moreover, because l_{ext} is smaller than the phantom thicknesses, these samples are confirmed to operate within the multiple-scattering regime, as expected from previous works [7, 14-15].

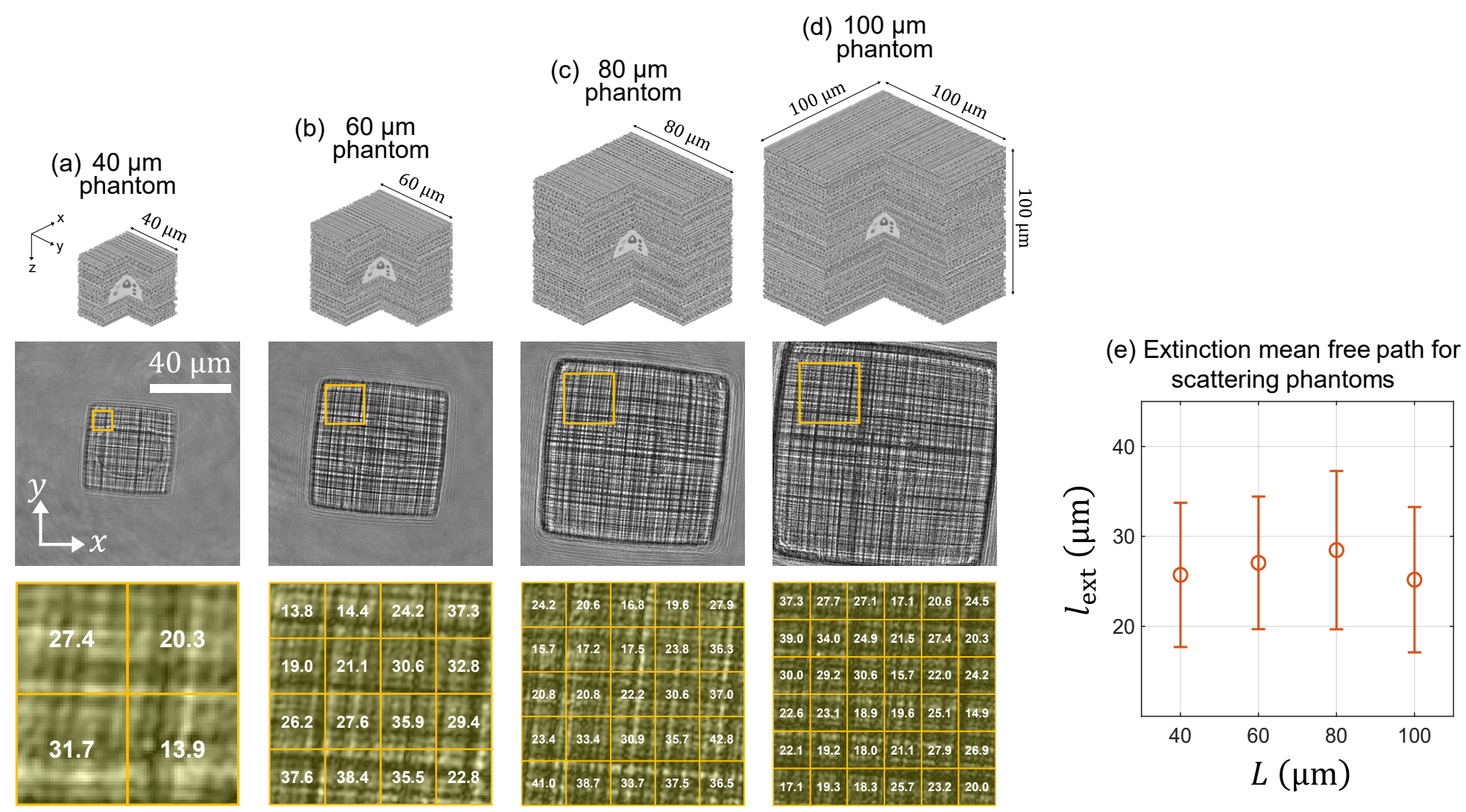

Figure S8.1 Extinction mean-free-path (l_{ext}) is characterized for scattering phantoms with thicknesses of L = **(a)** 40, **(b)** 60, **(c)** 80, and **(d)** 100 µm. The second row shows the measured scattering amplitude under normal (orthogonal) illumination. The yellow rectangles indicate the regions selected for analysis. Each selected region was subdivided into adjacent subregions, and l_{ext} was computed independently for each subregion, as shown in the third row. **(e)** Distribution of l_{ext} for each phantom thickness is shown. All four phantoms exhibit consistent distributions for l_{ext}, with an average value of approximately 25 µm and a standard deviation of approximately 7.5 µm.

We next applied the proposed framework to the multiple-scattering succulent and 48 hpf zebrafish embryo datasets presented in the main manuscript. Following the same procedure used for the scattering phantoms, we selected regions of interest within each sample that visually exhibited multiple scattering and subdivided each region into adjacent subregions. Each subregion was chosen to be sufficiently large compared to the underlying nano/microscale structural heterogeneity, while remaining small enough to provide a representative spatial distribution of extinction mean free paths for statistical analysis. For both the succulent and zebrafish embryo, we analyzed two anatomically separated scattering regions to investigate how l_{ext} varies across different tissue types within a large biological specimen. The results are shown in **Figure S8.2** below.

As shown, the l_{ext} varies substantially both between and within the biological samples. The succulent exhibited average l_{ext} of approximately 30 µm (FOV 1) and 35 µm (FOV 2), indicating moderate spatial variation across the tissue. The zebrafish embryo exhibited even greater heterogeneity, with FOV 1 (yolk region) yielding an average l_{ext} of approximately 25 µm, whereas FOV 2 (spinal cord region) yielded a larger value of approximately 33 µm. This

is expected because the spinal cord region is known to be less scattering than the yolk region. These results demonstrate that l_{ext} can vary considerably across different anatomical regions within the same biological specimen, reflecting underlying differences in tissue composition and microstructure. Importantly, in every case l_{ext} remained well below the corresponding sample thickness (succulent: $L = 131.6$ µm; zebrafish embryo: $L = 111.22$ µm). This confirms that all the analyzed regions reside within the multiple-scattering regime, even though the strength of scattering varies between specific regions.

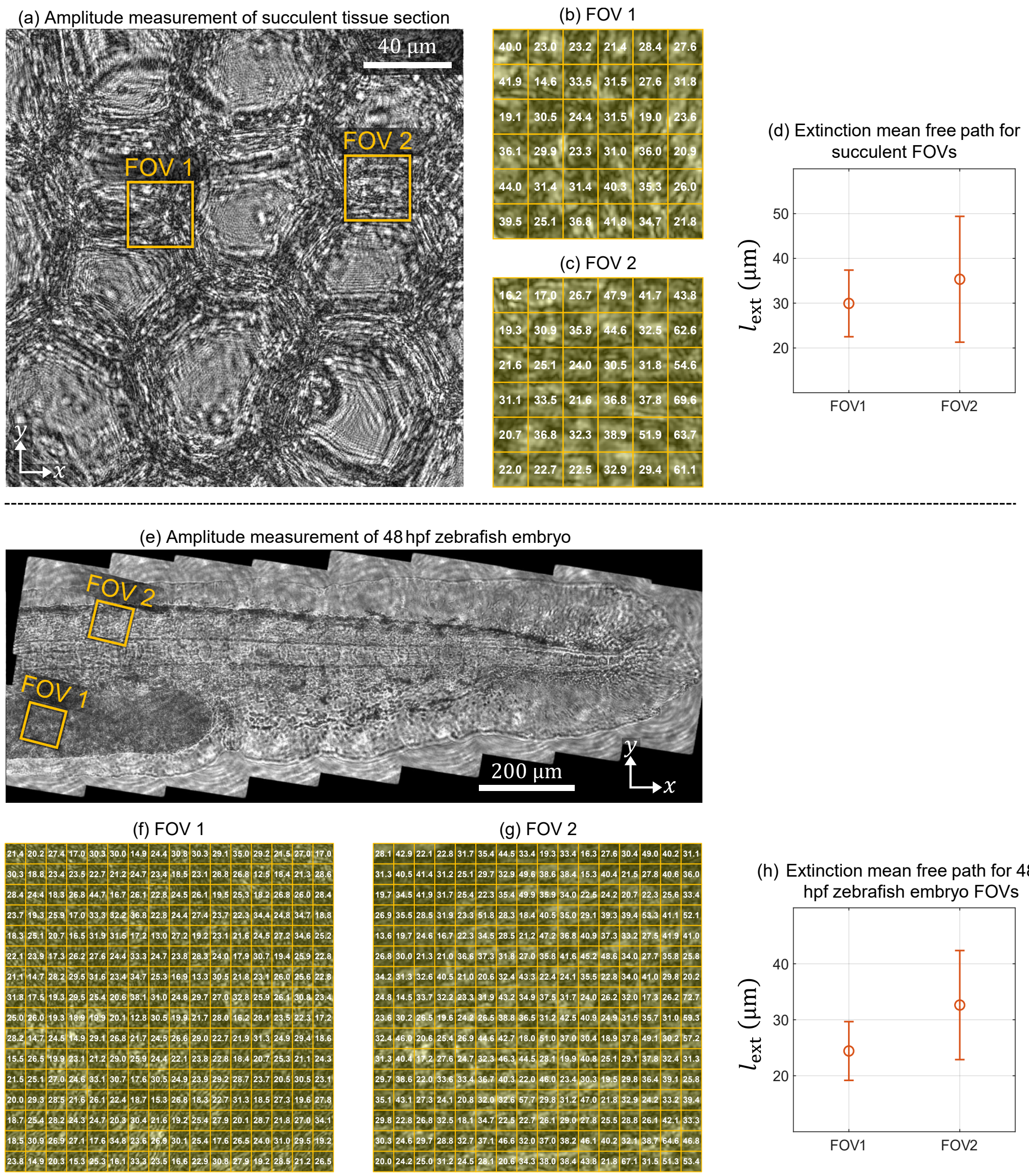

Figure S8.2. Quantification of extinction mean free path in biological samples. **(a)** Amplitude measurement of the succulent tissue section. Two representative fields of view (FOVs) were selected for analysis. **(b,c)** Each FOV was partitioned into adjacent subregions, and the extinction mean free path (l_{ext} in µm) was computed independently for each subregion. **(d)** Distribution of l_{ext} values for the two succulent FOVs. **(e)** Amplitude measurement of a 48 hpf zebrafish embryo with two representative FOVs selected for analysis. **(f,g)** Corresponding subregions and computed l_{ext} values. **(h)** Distribution of l_{ext} values for the two zebrafish FOVs.

S9. Comparing RI reconstruction results from weak-scattering Rytov versus MSBP multiple-scattering model

To illustrate the importance of accounting for multiple scattering, we compared our MSBP-based inverse-scattering reconstructions with reconstructions obtained using the weak-scattering Rytov approximation. The Rytov method is a widely used linear RI-reconstruction approach and assumes that multiple-scattering effects can be neglected. Under this approximation, the measured complex field is converted into a complex phase through the Rytov transform, mapped into three-dimensional Fourier space using the Fourier diffraction theorem, and synthesized into the volumetric RI distribution through filtered backpropagation. In this work, Rytov reconstructions were performed using the implementation described by Sung *et al.* [16].

Figure S9.1 below compares Rytov and MSBP reconstructions for the three biological samples shown in the main manuscript arranged in order of increasing scattering strength – unicellular alga (*Micrasterias radiata*), a succulent tissue section, and a 48 hpf zebrafish embryo. The alga represents a relatively thin and weakly scattering specimen, whereas the succulent tissue and zebrafish embryo are substantially thicker and exhibit pronounced multiple scattering.

For the algal sample, both reconstruction methods recover similar overall morphology and internal structure. This is expected because the weak-scattering assumptions underlying the Rytov approximation are reasonably satisfied for this specimen. Minor differences are present, but both methods produce comparable RI distributions.

In contrast, the performance of the two approaches diverges dramatically for the thicker biological specimens. For both the succulent tissue section and the zebrafish embryo,

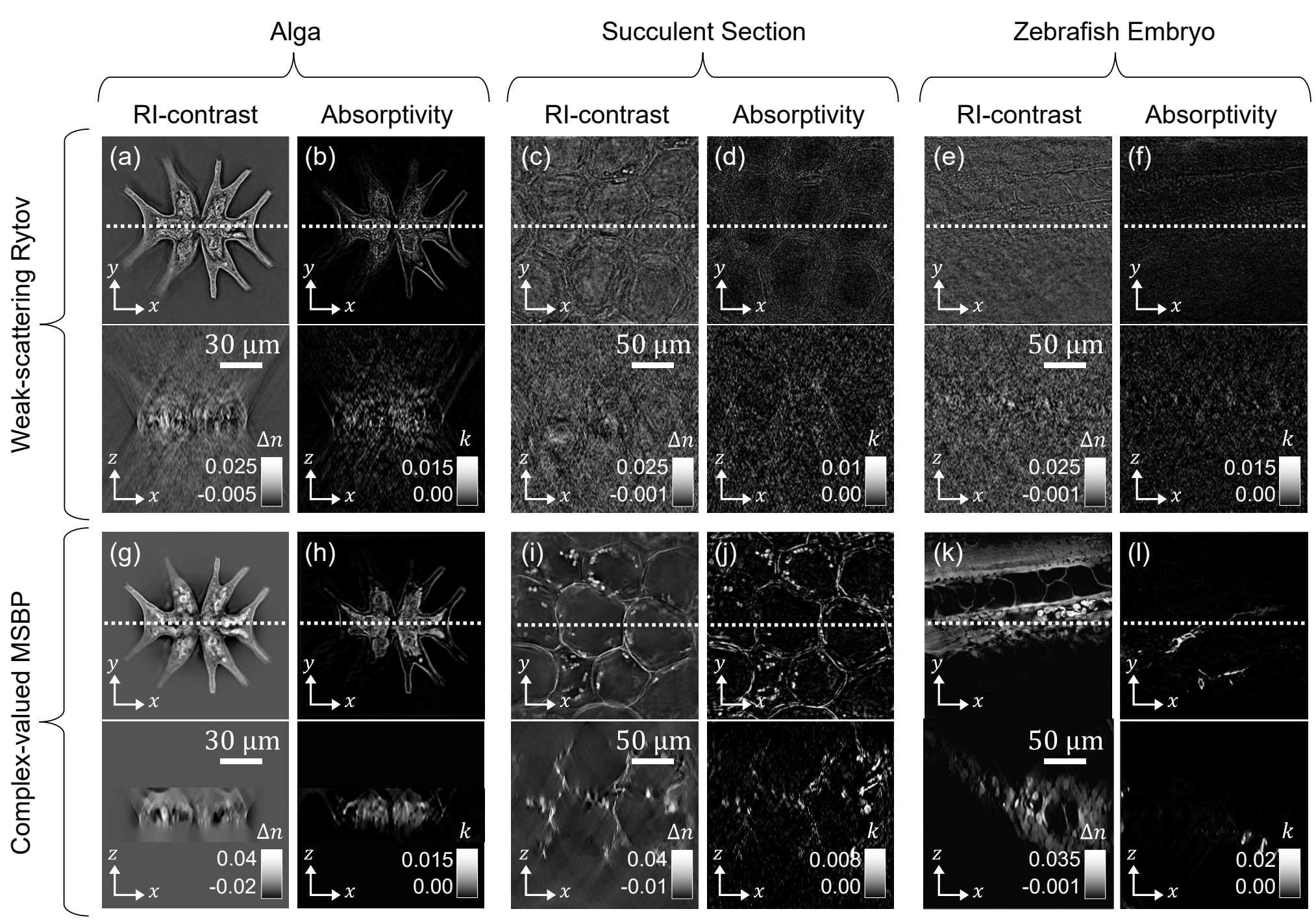

Figure S9.1. Comparison of 3D RI reconstructions obtained using the weak-scattering Rytov approximation and the multiple-scattering MSBP framework. **(a,b)** The Rytov approximation accurately reconstructs the morphology of the weakly scattering algae sample. For the more strongly scattering **(c,d)** succulent tissue section and **(e,f)** 48 hpf zebrafish embryo, the Rytov reconstruction fails to recover the underlying structure. **(g – l)** In contrast, the MSBP framework successfully reconstructs all three samples, demonstrating the importance of explicitly modeling multiple scattering in thick biological specimens.

the Rytov reconstruction exhibits almost no discernable features or image contrast, making it impossible to distinguish underlying morphology. The MSBP reconstructions, on the other hand, accurately recover the three-dimensional morphology of both samples. This demonstrates that while weak-scattering reconstruction methods remain effective for thin, weakly scattering specimens, accounting for multiple scattering becomes essential for accurately imaging thick biological tissues.

S10. Preparation and care of zebrafish embryo

Zebrafish embryos used in these experiments were raised to 48 hours post fertilization, staged according to Kimmel 1995 [17]. Embryos were fixed in 4% paraformaldehyde and placed at 4-degrees Celsius overnight. The next day embryos were dehydrated through a series of increasing concentrations of methanol in phosphate buffered saline (PBS), to 100% methanol. Embryos were then stored at -20-degrees Celsius. In preparation for imaging, embryos were rehydrated in the PBS:methanol series to 100% PBS.

All zebrafish were housed at UT Austin under the IACUC-approved protocol AUP-2023-00297 in the Eberhart lab vivarium. Zebrafish were raised according to (Westerfield 1993) [18].

S11. References

[1] K. Kreutz-Delgado, “The Complex Gradient Operator and the CR-Calculus,” Jun. 26, 2009, arXiv: arXiv:0906.4835. doi: 10.48550/arXiv.0906.4835.

[2] S. Chowdhury et al., “High-resolution 3D refractive index microscopy of multiple-scattering samples from intensity images,” Optica, OPTICA, vol. 6, no. 9, pp. 1211–1219, Sep. 2019, doi: 10.1364/OPTICA.6.001211.

[3] U. S. Kamilov et al., “Optical Tomographic Image Reconstruction Based on Beam Propagation and Sparse Regularization,” IEEE Transactions on Computational Imaging, vol. 2, no. 1, pp. 59–70, 2016, doi: 10.1109/TCI.2016.2519261.

[4] “Autograd mechanics — PyTorch 2.10 documentation.” Accessed: Feb. 27, 2026. [Online]. Available: https://docs.pytorch.org/docs/stable/notes/autograd.html

[5] R. Eckert, Z. F. Phillips, and L. Waller, “Efficient illumination angle self-calibration in Fourier ptychography,” Appl. Opt., AO, vol. 57, no. 19, pp. 5434–5442, Jul. 2018, doi: 10.1364/AO.57.005434.

[6] Y. Nesterov, Introductory Lectures on Convex Optimization, 1st ed., vol. 87. in Applied Optimization, vol. 87. Springer New York, NY.

[7] J. Kim et al., “Inverse-scattering in biological samples via beam-propagation,” Aug. 22, 2025, bioRxiv. doi: 10.1101/2025.08.17.670744.

[8] A. Beck and M. Teboulle, “Fast Gradient-Based Algorithms for Constrained Total Variation Image Denoising and Deblurring Problems,” IEEE Transactions on Image Processing, vol. 18, no. 11, pp. 2419–2434, Nov. 2009, doi: 10.1109/TIP.2009.2028250.

[9] J. Lim et al., “Comparative study of iterative reconstruction algorithms for missing cone problems in optical diffraction tomography,” Opt. Express, vol. 23, no. 13, pp. 16933–16948, Jun. 2015, doi: 10.1364/OE.23.016933.

[10] Y. Sung, W. Choi, C. Fang-Yen, K. Badizadegan, R. R. Dasari, and M. S. Feld, “Optical diffraction tomography for high resolution live cell imaging,” Opt. Express, OE, vol. 17, no. 1, pp. 266–277, Jan. 2009, doi: 10.1364/OE.17.000266.

[11] S. Chowdhury, W. J. Eldridge, A. Wax, and J. Izatt, “Refractive index tomography with structured illumination,” Optica, OPTICA, vol. 4, no. 5, pp. 537–545, May 2017, doi: 10.1364/OPTICA.4.000537.

[12] J. Li et al., “Transport of intensity diffraction tomography with non-interferometric synthetic aperture for three-dimensional label-free microscopy,” Light Sci Appl, vol. 11, no. 1, p. 154, Jun. 2022, doi: 10.1038/s41377-022-00815-7.
[13] O. Yasuhiko and K. Takeuchi, “Bidirectional in-silico clearing approach for deep refractive-index tomography using a sparsely sampled transmission matrix,” Biomed. Opt. Express, BOE, vol. 15, no. 9, pp. 5296–5313, Sep. 2024, doi: 10.1364/BOE.524859.
[14] W. Krauze, A. Kuś, M. Ziemczonok, M. Haimowitz, S. Chowdhury, and M. Kujawińska, “3D scattering microphantom sample to assess quantitative accuracy in tomographic phase microscopy techniques,” Sci Rep, vol. 12, no. 1, p. 19586, Nov. 2022, doi: 10.1038/s41598-022-24193-7.
[15] J. Kim and S. Chowdhury, “Space-time inverse-scattering of translation-based motion,” 2024, [Online]. Available: https://arxiv.org/abs/2412.09403
[16] Y. Sung, W. Choi, C. Fang-Yen, K. Badizadegan, R. R. Dasari, and M. S. Feld, “Optical diffraction tomography for high resolution live cell imaging,” Opt. Express, OE, vol. 17, no. 1, pp. 266–277, Jan. 2009, doi: 10.1364/OE.17.000266.
[17] C. B. Kimmel, W. W. Ballard, S. R. Kimmel, B. Ullmann, and T. F. Schilling, “Stages of embryonic development of the zebrafish,” Developmental Dynamics, vol. 203, no. 3, pp. 253–310, 1995, doi: 10.1002/aja.1002030302.
[18] M. Westerfield, The Zebrafish Book. A Guide for the Laboratory Use of Zebrafish (Danio rerio), 5th Edition. University of Oregon Press, Eugene, 2007.
